# PRIMAL: Page Rank-Based Indoor Mapping and Localization Using Gene-Sequenced Unlabeled WLAN Received Signal Strength

**DOI:** 10.3390/s151024791

**Published:** 2015-09-25

**Authors:** Mu Zhou, Qiao Zhang, Kunjie Xu, Zengshan Tian, Yanmeng Wang, Wei He

**Affiliations:** 1 Chongqing Key Lab of Mobile Communications Technology, Chongqing University of Posts and Telecommunications, Chongqing 400065, China; E-Mails: zhangqiao6120@gmail.com (Q.Z.); tianzs@cqupt.edu.cn (Z.T.); 2012210036@stu.cqupt.edu.cn (Y.W.); hewei@cqupt.edu.cn (W.H.); 2 Ericsson, San Jose, CA 95134, USA; E-Mail: xu.kunjie@gmail.com

**Keywords:** indoor mapping and localization, Allen logic, gene sequencing, graph drawing, page rank

## Abstract

Due to the wide deployment of wireless local area networks (WLAN), received signal strength (RSS)-based indoor WLAN localization has attracted considerable attention in both academia and industry. In this paper, we propose a novel page rank-based indoor mapping and localization (PRIMAL) by using the gene-sequenced unlabeled WLAN RSS for simultaneous localization and mapping (SLAM). Specifically, first of all, based on the observation of the motion patterns of the people in the target environment, we use the Allen logic to construct the mobility graph to characterize the connectivity among different areas of interest. Second, the concept of gene sequencing is utilized to assemble the sporadically-collected RSS sequences into a signal graph based on the transition relations among different RSS sequences. Third, we apply the graph drawing approach to exhibit both the mobility graph and signal graph in a more readable manner. Finally, the page rank (PR) algorithm is proposed to construct the mapping from the signal graph into the mobility graph. The experimental results show that the proposed approach achieves satisfactory localization accuracy and meanwhile avoids the intensive time and labor cost involved in the conventional location fingerprinting-based indoor WLAN localization.

## 1. Introduction

Nowadays, people spend more than 80% of their time in the indoor environment, where the signal from the Global Positioning System (GPS) is generally difficult to receive. In this circumstance, many indoor localization systems are proposed to guarantee the performance of a variety of location-based services (LBSs), like the guidance of shopping routes, security and healthcare for the elderly, and asset management in warehouses and modern buildings. At the same time, various kinds of techniques have been developed for indoor localization in the recent decade, like Bluetooth [1], ultrasonic wave [2], radio-frequency ID (RFID) [3], ultra-wideband (UWB) [4], visible light communications (VLC) [5,6,7] and wireless local area networks (WLAN) [8,9,10,11]. Among them, the received signal strength (RSS)-based WLAN localization technique is preferred due to the rapid development of WLAN infrastructures and mobile devices, as well as the wide deployment of WLAN, which is selected as one of the primary high-speed access networks in the indoor environment. To the best of our knowledge, trilateration and location fingerprinting are recognized as two of the most representative approaches used in RSS-based indoor WLAN localization. The performance of the trilateration approach suffers from the inaccurate estimation of the distance from each access point (AP) to the receiver [12,13]. The first reason is that the propagation models used for distance estimation cannot always be effective due to the irregular variation of RSS caused by the signal reflection, scattering and diffraction. The second reason is that many indoor areas where the APs are actually located are not reachable. In this case, both the AP and target locations should be estimated simultaneously, and thereby, the precision of distance estimation generally drops dramatically. On the contrary, the location fingerprinting approach is preferred [14,15,16,17]. This approach consists of two phases. In the offline phase, the RSSs at a batch of pre-calibrated reference points (RPs) with known physical coordinates are collected and then stored as the location fingerprints into the radio map. In the online phase, the newly-collected RSSs are matched against the radio map to realize the location estimation. However, the time and labor cost involved in the radio map construction increases rapidly as the area increases.

To solve the cost problem and to guarantee the accuracy of RSS-based indoor WLAN localization, we propose a novel page rank-based indoor mapping and localization (PRIMAL) by using the gene-sequenced unlabeled WLAN RSS for simultaneous localization and mapping (SLAM). In concrete terms, we first carry out the observation of the motion patterns of the people in the target environment to construct the mobility graph by using the Allen logic. Second, we rely on off-the-shelf smartphones to collect the WLAN RSSs, which are not labeled with physical coordinates. Third, the concept of gene sequencing is adopted to determine the correlation relations among different RSS sequences, so as to assemble the RSS sequences into a signal graph. Fourth, we utilize the graph drawing approach to exhibit the graphs in a more readable manner. Finally, by using the proposed page rank (PR) algorithm, the mapping from the signal graph into the mobility graph is constructed. After the previous steps, the receiver can be located in the area mapped from the node that is matched by the newly-collected RSSs in the signal space.

The rest of this paper is organized as follows. In Section 2, we show some related work on the existing SLAM approaches in the indoor WLAN environment. The steps of the proposed PRIMAL are discussed in detail in Section 3. The extensive experimental results are provided in Section 4. Finally, Section 5 concludes the paper and presents some future directions.

## 2. Related Work

In response to the cost problem faced by location fingerprinting in the conventional RSS-based indoor WLAN localization [18,19,20,21], many existing works suggested using motion sensing as a candidate to perform the localization. The authors in [22] collected the WLAN RSSs to construct a logic graph, which can be used to characterize the physical layout of the target environment, and meanwhile rely on an accelerator to explore the reachability among different physical areas, as well as to detect the status of RSSs. After that, based on the constructed mapping relationship between the logic graph and ground-truth graph, the target location is estimated in a specific area for each location query. Using a smartphone, the authors in [23] invented a pedestrian tracking system, which can automatically construct both the floor plan of the anonymous target environment and the corresponding radio map. An indoor tracking system based on the labeled topological map constructed by SLAM is addressed in [24]. See [25]: a foot-mounted inertial measurement unit (IMU) is used to perform proprioceptive motion sensing, and meanwhile, an action recognition system is applied to observe the landmarks of location-related actions. The authors in [26] proposed a pedestrian tracking system by integrating the odometry data collected by the foot-mounted IMU and WLAN RSSs. The localization system developed in [27] is based on the fusion of the image data and data from the IMU in a smartphone. The GraphSLAM approach proposed in [28] is appropriate for a large-scale environment, since there is no signature uniqueness assumption in the GraphSLAM. The authors in [29] constructed a multi-modal signal map from the RSSs collected by all of the available sensors. The work in [30] depended on the IMU sensors to label the RSSs as the pedestrian walks in the same direction. A new concept of the Wi-Fi fingerprint (FP), which considers the order relation among the RSS rather than the absolute values of RSSs, is addressed in [31]. The authors in [32] present a new localization approach, in which the training data are obtained by means of finite difference time domain (FDTD) simulations of electromagnetic propagation.

To deal with the computational complexity problem for localization, the authors in [33] use decision trees to minimize the complexity of the localization system. The authors in [34] rely on the joint clustering technique, which performs the clustering of locations to reduce the computational cost. A new low-complexity tracking scheme is proposed in [35], which is based on Fano’s sequential decoding algorithm. The authors in [36] propose the multiple filters (MFs)-based implementation approach, which achieves a significant reduction of the computational complexity. The authors in [37] compare the performance of the probabilistic Gaussian kernel fingerprint-based indoor positioning algorithm by using different types of smartphones. An energy-efficient WLAN-based indoor positioning algorithm, which factors out every part of the probabilistic fingerprint formulae, is proposed in [38].

Different from the existing work in the literature, we propose a novel indoor mapping and localization approach, namely the PRIMAL, which is independent of location fingerprinting and motion sensing. Furthermore, there is no requirement of extra infrastructure or devices compared to the conventional approaches. The four main contributions of this paper are summarized as follows. First of all, there is no requirement of location fingerprinting and motion sensing, which saves much time and labor cost. Second, based on people’s motion pattern observation, the mobility graph, which is constructed by using the Allen logic, can help greatly in investigating the motion behavior of the people in the target environment. Third, we apply the graph drawing approach to exhibit both the mobility and signal graphs in a more readable manner. Finally, by adopting the PAalgorithm, we conduct the indoor mapping and localization simultaneously. Table 1 summarizes the main symbols used in this paper.

**Table 1:** Symbol notation.

Symbols	Description
$I_{z}$	Time duration of the event *z*
$a_{i}$	The *i*-th RSS vector in sequence *a*
$b_{j}$	The *j*-th RSS vector in sequence *b*
$H\left(i,j\right)$	Matching score between $a_{i}$ and $b_{j}$
*m*	Length of sequence *a*
*n*	Length of sequence *b*
$s(a_{i},b_{j})$	Similarity function of RSS pair $a_{i}$ and $b_{j}$
$W_{k}$	Gap scoring function with depth *k*
${\mathrm{RSS}}_{l}$	The *l*-th RSS sequence
${\mathrm{rss}}_{li}$	The *i*-th RSS vector in ${\mathrm{RSS}}_{l}$

## 3. System Description

### 3.1. Construction of the Mobility Graph

**Figure 1:**
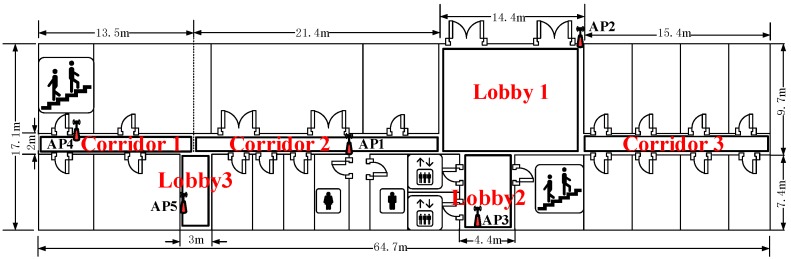
Layout of the target area.

**Figure 2:**
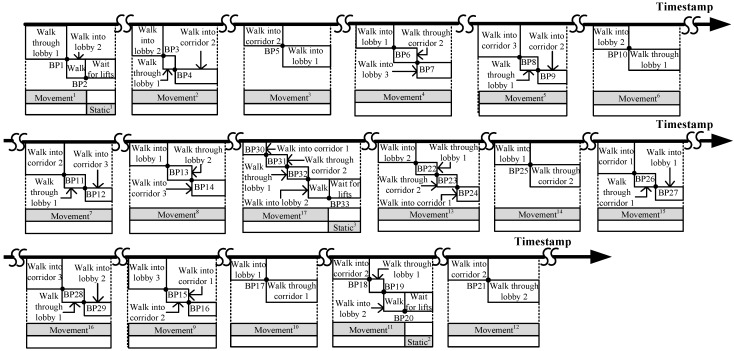
Motion patterns.

To obtain the connectivity among different areas of interest, we conduct the people’s motion pattern observation in the target environment. Figure 1 shows the layout of the six areas of interest, and Figure 2 illustrates the people’s 17 motion patterns during a working day. Each motion pattern consists of different events, which are separated by the break points (BPs).

**Figure 3:**
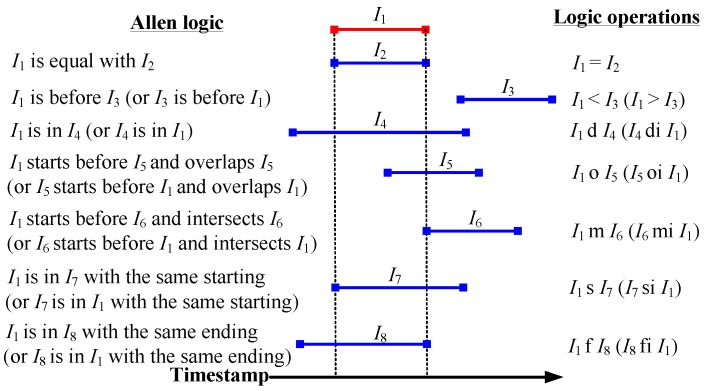
The Allen logic for event expression.

**Figure 4:**
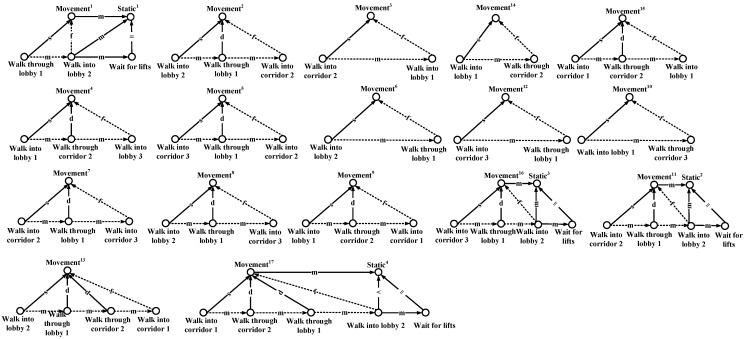
Event graphs.

**Figure 5:**
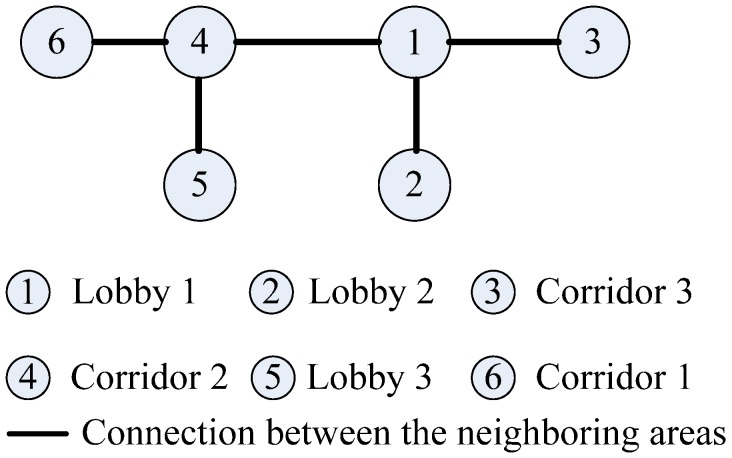
Mobility graph.

In the Allen logic, the relations of events can be represented by 13 different logic operations, as described in Figure 3. On this basis, by using the Allen logic, we draw the event graphs corresponding to the people’s 17 different motion patterns in Figure 4. In each graph, we mark the longest path (or the path involving the largest number of hops) with a dashed line. On each longest path, the event nodes are connected by the edges with the “m” operation, while the last even node and movement node are connected by an edge with the “f” operation. The event nodes that are connected by the edges with the “m” operation indicate the adjacent events happening in chronological order, while the edge with the “f” operation connecting the final event node and movement node indicates that the last event corresponds to the movement state. Based on this, the event nodes on the longest path can be used to describe the connectivity among different areas of interest. For instance, in the first graph, based on the longest path that is associated with the movement ${}^{1}$, we obtain a motion behavior between Lobby 1 and Lobby 2 and thereby construct a connection between these two lobbies. Hence, the mobility graph can be constructed by considering all of the motion behaviors of the people in the target environment, as shown in Figure 5.

### 3.2. Construction of the Signal Graph

#### 3.2.1. RSS Characteristics

**Figure 6:**
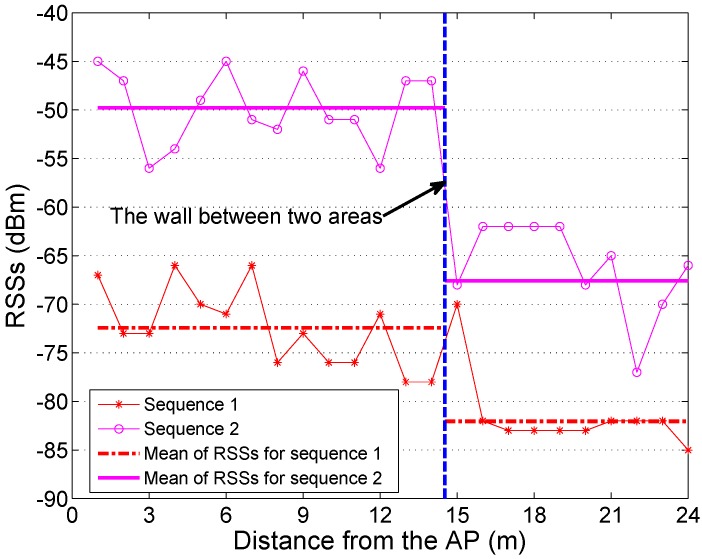
Variation of WLAN RSSs.

A significant reason for using the WLAN RSSs to conduct the SLAM is due to the property that the WLAN RSSs collected in two different areas that are separated by a wall could vary greatly. Figure 6 shows an example of the variations of two WLAN RSS sequences as the signal goes through a wall. We take Sequence 1 (with *’s) as an example. The mean of the RSS before crossing the wall is about −72 dBm, whereas after crossing the wall, it decreases to −82 dBm. Hence, the variation of WLAN RSSs can help with characterizing the layout of the target environment.

#### 3.2.2. Gene Sequencing

**Figure 7:**
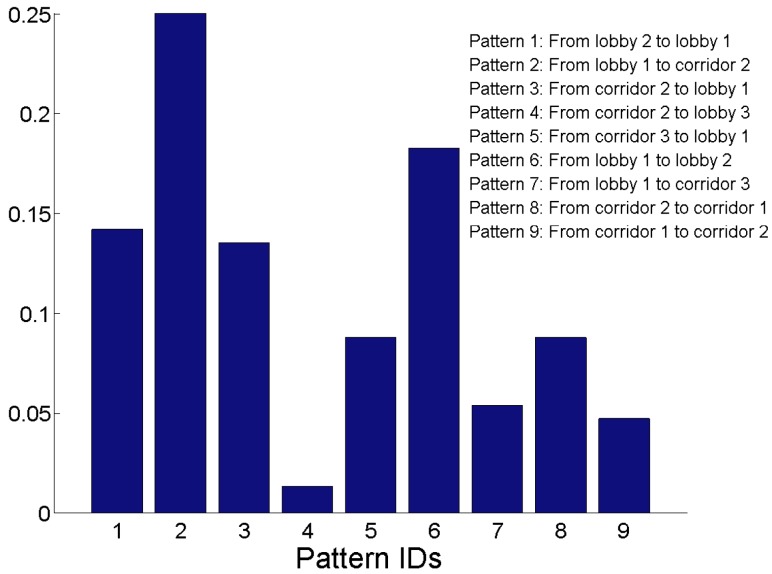
Frequencies of area transitions.

**Figure 8:**
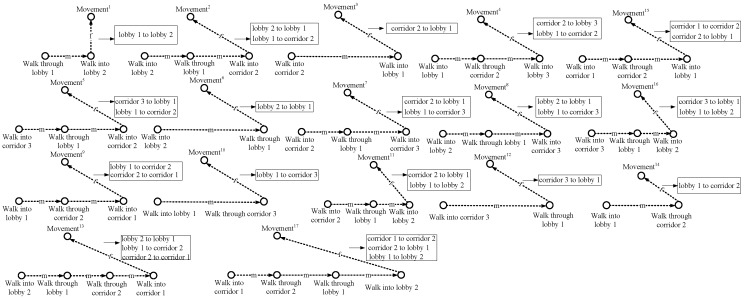
Path separation.

Based on the people’s motion pattern observation during a working day, we obtain the frequencies of the area transitions between every two adjacent areas of interest in Figure 7. There are in total nine patterns of area transitions counted by the path separation, as shown in Figure 8.

**Figure 9:**
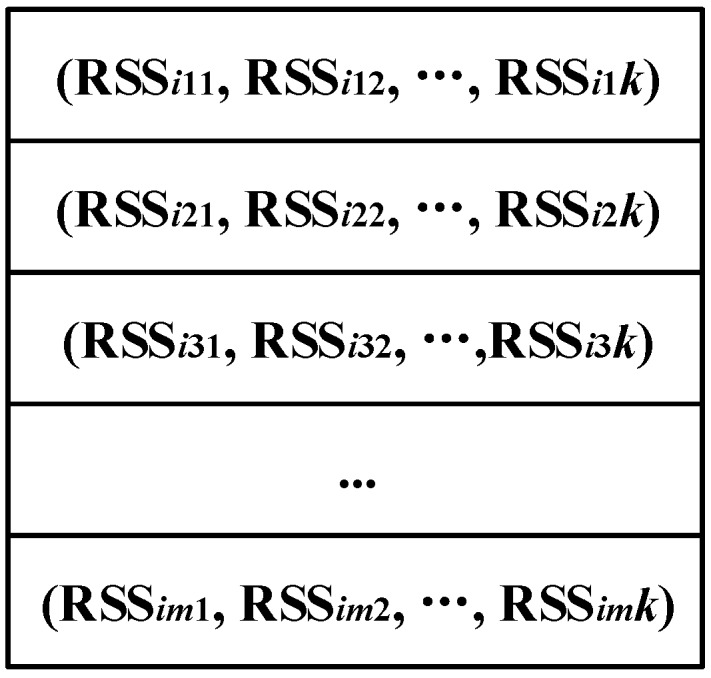
Format of RSS sequences.

In the target environment, we collected 94 WLAN RSS sequences that obey the frequencies of area transitions shown in Figure 7. Figure 9 illustrates the format of RSS sequences where *k* is the number of APs, ${\mathrm{RSS}}_{ijl}$ is the *j*-th RSS vector in the *i*-th RSS sequence from the *l*-th AP and *m* is the number of RSS vectors in each sequence. Table 2 shows the number of RSS sequences collected on each trace. Hence, the collected RSS sequences can not only reflect the connectivity among different areas of interests, but also depict the motion patterns of the people in the signal space. Therefore, by separating each RSS sequence into different segments of RSSs with high correlation, we can obtain the transitions of segments and consequently assemble the RSS sequences into a signal graph.

**Table 2:** Number of RSS sequences on each trace.

Traces	Number of Collected RSS Sequences
Lobby 2 → Lobby 1	10
Lobby 2 → Corridor 2	5
Corridor 2 → Lobby 1	7
Lobby 1 → Corridor 2 → Lobby 3	2
Corridor 3 → Corridor 2	9
Lobby 1 → Lobby 2	19
Corridor 2 → Corridor 3	4
Lobby 2 → Corridor 3	1
Lobby 1 → Corridor 2 → Corridor 1	8
Lobby 1 → Corridor 3	3
Corridor 2 → Lobby 2	2
Corridor 3 → Lobby 1	2
Lobby 2 → Corridor 2 → Corridor 1	5
Lobby 1 → Corridor 2	8
Corridor 1 → Corridor 2 → Lobby 1	3
Corridor 3 → Lobby 2	2
Corridor 1 → Corridor 2 → Lobby 2	4

In gene sequencing, given two sequences, *a* and *b*, a scoring matrix, *H*, is constructed to detect the segments of RSSs with high correlation, namely the correlation segments, between these two sequences. The elements in *H* are calculated by:

(1) $$\begin{array}{c} H(i,0)=0,0\leq i\leq m,H(0,j)=0,0\leq j\leq n\\ H\left(i,j\right)=\max\left\{\begin{array}{c} 0\\ H\left(i-1,j-1\right)+s\left(a_{i},b_{j}\right)\\ \max_{k\geq 1}\left\{H\left(i-k,j\right)+W_{k}\right\}\\ \max_{l\geq 1}\left\{H\left(i,j-l\right)+W_{l}\right\} \end{array}\right\}\\ 1\leq i\leq m,1\leq j\leq n \end{array}$$

where H(i,j) is the matching score between the *i*-th nucleotide in *a*, $a_{i}$ and the *j*-th nucleotide in *b*, $b_{j}$. Using this concept, in our system, we view the WLAN RSS sequences as the gene sequences, *i.e.*, $a∼{\mathrm{RSS}}_{l}=\left\{{\mathrm{rss}}_{l1},{\mathrm{rss}}_{l2},\ldots {\mathrm{rss}}_{lm}\right\}$ and $b∼{\mathrm{RSS}}_{i}=\left\{{\mathrm{rss}}_{i1},{\mathrm{rss}}_{i2},\ldots \;{\mathrm{rss}}_{in}\right\}$, and the RSS vectors as the nucleotides, *i.e.*, $a_{i}∼{\mathrm{rss}}_{li}$ and $b_{j}∼{\mathrm{rss}}_{ij}$. Thus, the calculation of the matching score between the RSS vectors equals the one between the corresponding nucleotides. To detect the correlation segments, we require that the matching scores between the RSS vectors satisfy:

- H(i,j)≥H(i,j+1) as $a_{i}=b_{j}$ and $a_{i}\neq b_{j+1}$
- H(i,j)≥H(i+1,j+1) as $a_{i}=b_{j}$ and $a_{i+1}\neq b_{j+1}$
- H(i,j)≤H(i+1,j+1) as $a_{i}=b_{j}$ and $a_{i+1}=b_{j+1}$
- H(i,j)≥H(i+1,j+1) as $a_{i}\neq b_{j}$ and $a_{i}\neq b_{j+1}$
- H(i,j)≤H(i+1,j+1) as $a_{i}\neq b_{j}$ and $a_{i+1}=b_{j+1}$

These requirements indicate the characteristics as follows.

(i) If the current RSS vector is matched with an RSS vector, but mismatched with the next one, the matching score of the current RSS pair is not lower than the one of the next pair;
(ii) if the current RSS pair is matched, whereas the next RSS pair is mismatched, the matching score of the current RSS pair is not lower than the one of the next pair;
(iii) if the current RSS pair is matched, while the next RSS pair is also matched, the matching score of the current RSS pair is not higher than the one of the next pair;
(iv) if the current RSS vector is mismatched and still mismatched with the next one, the matching score of the current RSS pair is not lower than the one of the next pair;
(v) if the current RSS pair is mismatched, whereas the next RSS pair is matched, the matching score of the current RSS pair is not higher than the one of the next pair.

**Figure 10:**
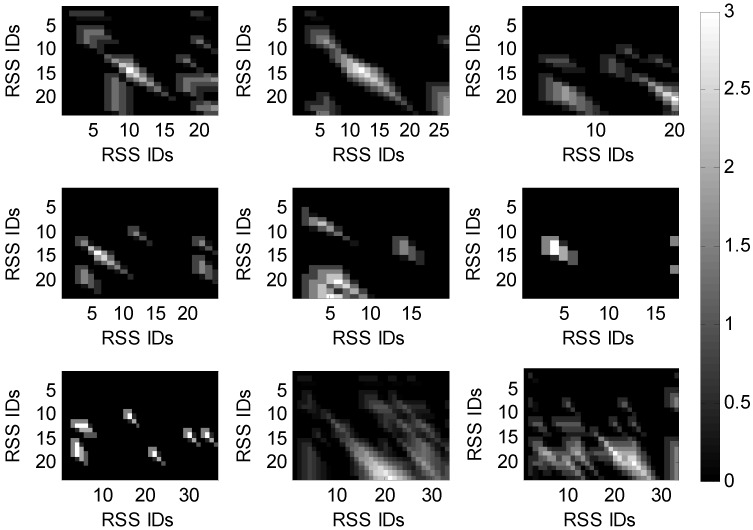
Scoring matrices.

To satisfy these requirements, we define the similarity function, $s(a_{i},b_{j})$, and gap scoring function, $W_{k}$, in Equation (2). Based on this, the matching scores satisfy the previous requirements, as proven in the Appendix Section. For the collected 94 RSS sequences, we construct in total 4371 scoring matrices (see Supplementary Material). Figure 10 shows nine of them in which each pixel represents the matching score of an RSS pair, while the larger pixel values indicate the higher similarities between the RSS vectors.

(2) $$\left\{\begin{array}{c} s\left(a_{i},b_{j}\right)=\left\{\begin{array}{c} \text{α}>0,a_{i}=b_{j}\\ \text{β}<0,a_{i}\neq b_{j} \end{array}\right.\\ W_{k}=-\left(\text{α}-\text{β}\right)k \end{array}\right.$$

where α and β stand for the reward score and penalty score, respectively.

To detect the correlation segments between two RSS sequences, we start from the highest matching score in the corresponding scoring matrix. The steps of this process are described as follows.

Step 1: Locate the highest score in *H*, notated as H(i,j), and then store the location (i,j) into the set *L*;

Step 2: Set H(i,j)=max{H(i-1,j),H(i,j-1),H(i-1,j-1)};

Step 3: Repeat Steps 1 and 2 until we obtain H(i,j)=0. We notate L(r)(1≤r≤gb) as the *r*-th location in *L*, and gb is the number of locations stored in *L*;

Step 4: Set r=gb;

Step 5: Examine the jump relation between the locations L(r) and L(r-1). If there is a diagonal jump [39] from L(r) to L(r-1), the vertical and horizontal coordinates of L(r-1) are selected to indicate the IDs of the RSS pair with high correlation;

Step 6: Set r=r-1;

Step 7: Repeat Steps 5 and 6 until *r* decreases to two.

**Figure 11:**
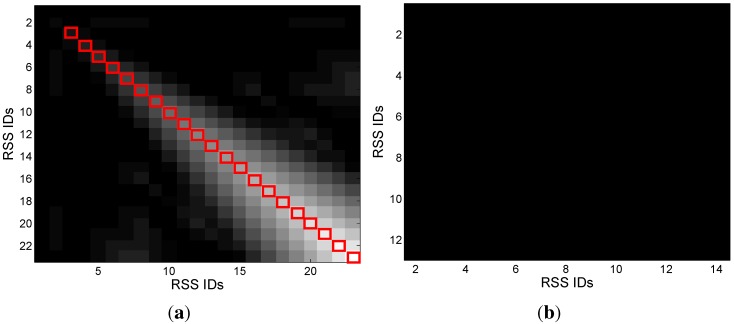
(**a**) A pair of the same RSS sequences; (**b**) a pair of different RSS sequences. Scoring matrices with respect to the same and different RSS sequences.

**Figure 12:**
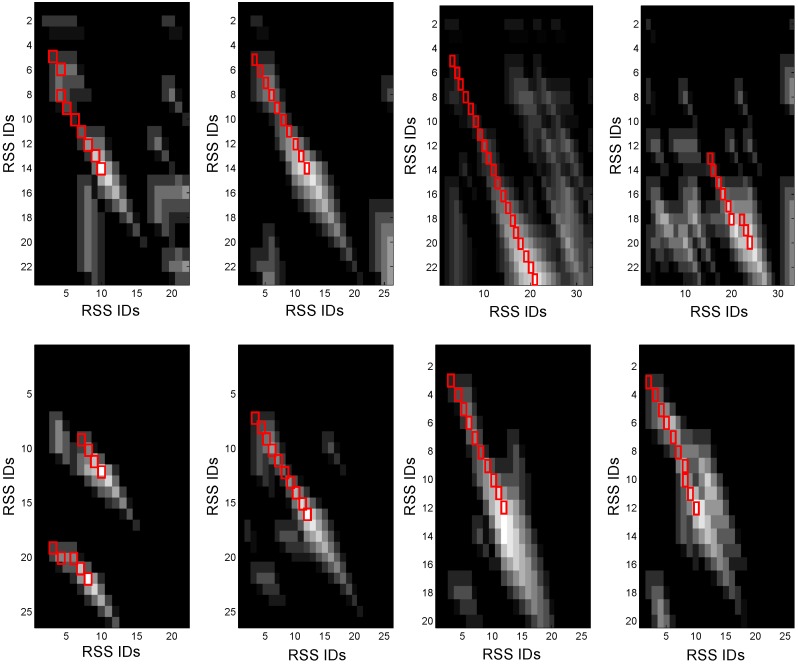
Result of diagonal jumps.

Figure 11 gives an example of the scoring matrices with respect to a pair of the same RSS sequences and a pair of RSS sequences collected in different areas, respectively. Obviously, based on the result of the diagonal jumps, which are marked with red rectangles in Figure 12, we detect the segments with high correlation. Hence, we can combine the RSS sequences into different clusters, while the RSSs in the same cluster are featured with high correlation.

**Figure 13:**
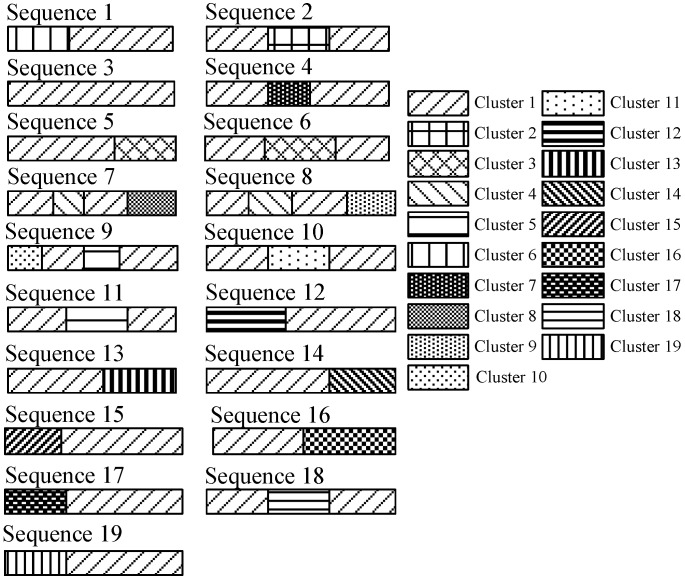
Result of RSS sequence combination.

**Figure 14:**
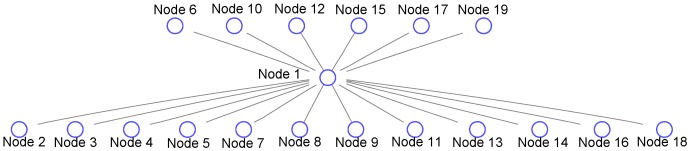
Signal graph.

Figure 13 shows the result of RSS sequence combination. There are in total 19 clusters. Based on the transition relations of different clusters, we can assemble the RSS sequences into a signal graph in which each node represents a cluster, while each edge represents the transition relation between two neighboring clusters. The signal graph finally constructed by using the gene sequencing is shown in Figure 14.

### 3.3. Graph Exhibition by Graph Drawing

In graph theory, during the visual exhibition of graphs, we can obtain many different layout structures with respect to a given graph, namely the isomorphic graphs. The readable, unique and clear graph exhibition can help greatly in exploring the relations between the graphs in the graph visualization aspect. To achieve this goal, we apply the graph drawing approach to guarantee the uniqueness of the layout structures of the mobility and signal graphs with the purpose of avoiding the confusion of the isomorphic graphs.

Specifically, give a undirected graph G=(V,E), we denote V=$\{v_{1},v_{2},$...,$v_{nd}\}$ and $E=\;\{...,e_{ij},...\}$ as the sets of vertices and edges. nd is the number of vertices, while $e_{ij}$ indicates that the vertices $v_{i}$ and $v_{j}\left(i,j\in \left\{1,2,...,nd\right\}\right)$ are connected by an edge. The steps of the process of graph drawing are described as follows.

Step 1: Construct the set of vertices, $V_{s}=\left\{\ldots ,v_{i},\ldots \right\}\left(i\in \left\{1,2,\ldots ,nd\right\}\right)$, in which the vertices have the same smallest degrees. The degree of vertex $v_{i}$, $d_{i}$, is defined as the number of vertices connected with $v_{i}$. If there are two vertices $v_{i}$ and $v_{j}$ in $V_{s}$ satisfying the relations that for any vertex $v_{r}\left(r\in \left\{1,2,\ldots ,nd\right\}\right)$ in $V-\{v_{i},v_{j}\}$, there is at least one path connecting $v_{i}$ and $v_{j}$, *i.e.*, $v_{i}e_{ik}v_{k}\cdots v_{r}e_{rt}v_{t}\cdots v_{j}$, we denote $v_{i}$ and $v_{j}$ as $v_{s}$ and $v_{t}$, respectively, and the set of all the other vertices, $V_{s}-\left\{v_{i},v_{j}\right\}$, as $v_{s}^{\prime }$. Otherwise, we select another vertex with the smallest degree in $V-V_{s}$, $v_{t^{\prime }}$. Then, we denote $v_{i}$ and $v_{t^{\prime }}$ as $v_{s}$ and $v_{t}$, respectively, and the set $V_{s}-\left\{v_{i}\right\}$ as $v_{s}^{\prime }$.

Step 2: After the vertices $v_{s}$, $v_{s}^{\prime }$ and $v_{t}$ are obtained, we continue to construct a direct graph $G^{\prime }$ from *G*. In concrete terms, for each vertex $v_{i}\left(i\in \left\{1,2,\ldots ,nd\right\}\right)$ in $V-\{v_{s},v_{s}^{\prime },v_{t}\}$, we construct the set of all direct paths as $PA_{i}=\left\{\left(v_{s}e_{sy}v_{y}...e_{ri}v_{i}...v_{j}e_{jt}v_{t}\right)\cup \left(v_{s}^{\prime }e_{sy^{\prime }}v_{y^{\prime }}...e_{r^{\prime }i}v_{i}...v_{j^{\prime }}e_{j^{\prime }t}v_{t}\right)\cup ...\right\}=\left\{P_{a_{1}}\cup P_{a_{2}}\cup ...P_{a_{t}}\right\}\left(y,y^{\prime },r,r^{\prime },j,j^{\prime }\in \left\{1,2,...,nd\right\}\right)$ starting from $v_{s}$ (or $v_{s}^{\prime }$) to $v_{t}$ and containing $v_{i}$, where *t* is the number of paths in $PA_{i}$ and $P_{a_{j}}\left(j\in \left\{1,2,...,t\right\}\right)$ is the *j*-th path in $PA_{i}$. After that, the set of direct edges, $E_{D}$, can be easily obtained from $PA_{i}$. A direct edge $e_{ij}\in E_{D}$ exists as there is a path $P_{a_{j}}\in PA_{i}$ satisfying $e_{ij}\in P_{a_{j}}$.

Step 3: Label the vertices $v_{i}\left(i\in \left\{1,2,\ldots ,nd\right\}\right)$ in $G^{\prime }$, notated as number($v_{i}$), based on the criteria as follows. We set: (i) number($v_{s}$) =0, number($v_{s}^{\prime }$) =0 and number($v_{j}$) = number($v_{i}$) +1 for the vertices $v_{i}$ and $v_{j}$ on the direct edge $e_{ij}$; and (ii) number($v_{k}$) = number($v_{i}$) +1 and number($v_{j}$) = number($v_{k}$) +1 for $\left\{e_{ij},e_{ik},e_{kj}\right\}\subset E_{D}$.

Step 4: Eliminate the edges on $Pa_{1}$, which starts from $v_{s}$ (or $v_{s}^{\prime }$) to $v_{t}$ and passes by the largest number of edges from $G^{\prime }$. We repeat this process until all of the edges have been eliminated from $G^{\prime }$ or there is no remaining path starting from $v_{s}$ (or $v_{s}^{\prime }$) to $v_{t}$. If there are remaining edges after the elimination process, we recognize each remaining edge as a distinct path. After that, we continue to detect all of the internal faces of *G*, $\left\{F_{h}\right\}\left(h=1,2,...,\eta \right)$, where η is the number of internal faces. An internal face is defined as a closed region (or the region containing no edge) with the boundary consisting of the edges in the graph. The external face of *G*, *F*, has the boundary, *C*, containing all of the nodes in *G*, notated as $C=\left(e_{ij}v_{j}e_{jr}...v_{s}e_{sl}...v_{t}e_{tu}...e_{xy}v_{y}e_{yi}\right)\left(i,j,r,l,u,x,y\in \left\{1,2,...,nd\right\}\right)$. We divide *F* into two sub-regions, $F_{s}$ and $F_{t}$, where $F_{s}$ and $F_{t}$ satisfy the relation $F_{s}\cap F_{t}=\left\{v_{s},v_{t}\right\}$.

Step 5: Construct a undirected graph $U=(V_{U},E_{U})$, where $V_{U}$=${\left\{Pa_{i}\right\}}_{i=1}^{\lambda }$∪${\left\{F_{h}\right\}}_{h=1}^{\eta }$∪$\left\{F_{s},F_{t}\right\}$=$\left\{v_{U_{1}},v_{U_{2}},...,v_{U_{d}}\right\}$, $E_{U}$=$\left\{e_{ij}\right\}$∪$\left\{e_{lh}\right\}$(i,h∈$\{U_{\lambda +1}$, $U_{\lambda +2}$, ..., $U_{\lambda +\eta }$, $U_{\lambda +\eta +1}$, $U_{\lambda +\eta +2}\}$, j,l∈$\left\{U_{1},U_{2},...,U_{\lambda }\right\}$), and λ is the number of detected paths in Step 4. We notate the sets of vertices involved in ${\left\{Pa_{i}\right\}}_{i=1}^{\lambda }$, ${\left\{F_{h}\right\}}_{h=1}^{\eta }$ and $\left\{F_{s},F_{t}\right\}$ as $\left\{v_{U_{1}},v_{U_{2}},...,v_{U_{\lambda }}\right\}$, $\left\{v_{U_{\lambda +1}},v_{U_{\lambda +2}},...,v_{U_{\lambda +\eta }}\right\}$ and $\left\{v_{U_{\lambda +\eta +1}},v_{U_{\lambda +\eta +2}}\right\}$, respectively. We regard $Pa_{i}(1$≤i≤λ) as a path vertex, $v_{U_{j}}\left(1\leq j\leq \lambda \right)$. Similarly, $v_{U_{j}}\left(\lambda +1\leq j\leq \lambda +\eta +2\right)$ is regarded as a face vertex. The number of vertices in *U* equals d=λ+η+2. $e_{ij}$ determines whether there is an edge intersection between the boundaries of the faces of ${\left\{F_{h}\right\}}_{h=1}^{\eta }\cup \left\{F_{s},F_{t}\right\}$ and ${\left\{Pa_{i}\right\}}_{i=1}^{\lambda }$. For simplicity, we notate $v_{U_{\lambda +\eta +1}}$ and $v_{U_{\lambda +\eta +2}}$ as $v_{U_{s}}$ and $v_{U_{t}}$.

Step 6: Denote *U*, $v_{U_{s}}$ and $v_{U_{t}}$ as *G*, $v_{s}$ and $v_{t}$, respectively, and then, construct the direct graph $U^{\prime }=\left(V_{U^{\prime }},E_{U^{\prime }}\right)$ from $U=(V_{U},E_{U})$ based on Step 2.

Step 7: Label the vertices in $U^{\prime }$ based on the criteria as follows. We set: (i) number($v_{U_{s}}$) =-0.5 and number($v_{j}$) = number($v_{i}$) + 0.5 for the vertices $v_{U_{i}}$ and $v_{U_{j}}$(i,j∈{1,2,...,d}) on $e_{ij}$; and (ii) number($v_{k}$) = number($v_{i}$) +0.5 and number($v_{j}$) = number($v_{k}$) +0.5 for $\left\{e_{ij},e_{ik},e_{kj}\right\}\subset E_{U^{\prime }}$.

Step 8: Determine the coordinates of the vertices and edges in *G* based on the criteria as follows. (i) For each vertex, $v_{i}\left(1\leq i\leq nd\right)$, the *Y* coordinate is the assigned value of $v_{i}$ in *G*, while the range of *X* coordinates is from the minimum to maximum values assigned to the path associated with $v_{i}$ in $U^{\prime }$; and (ii) for each edge, $e_{ij}\left(i,j\in \left\{1,2,...,nd\right\}\right)$, the *X* coordinate is the assigned value of $e_{ij}$ in $U^{\prime }$, while the range of *Y* coordinates is from the minimum to maximum values assigned to the vertices associated with $e_{ij}$ in $G^{\prime }$.

**Figure 15:**
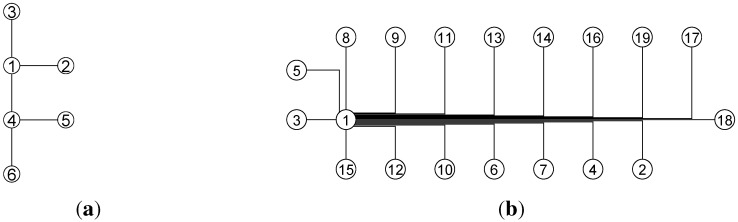
(**a**) Mobility graph; (**b**) signal graph. Results of graph drawing.

Figure 15 shows the results of graph drawing for the mobility graph and the signal graph, respectively. From this figure, we observe that by applying the graph drawing approach, the layout structure of graphs becomes unique and more readable in the visualization aspect.

### 3.4. Page Rank Algorithm

After the mobility and signal graphs are obtained, we propose to use the PR algorithm to construct the mapping from the signal graph into the mobility graph with the purpose of investigating the relation between the physical layout and signal distribution in the target environment.

First of all, based on the results of path separation in Figure 8, we focus on the detection of the hot areas, which appear frequently in the people’s motion patterns. To achieve this goal, we define $P_{z}\left(i\right)$ as the probability that the individual has visited the *i*-th area at the timestamp *z*. Thus, we have:

(3) $$P_{z+1}\left(i\right)=\sum_{j}P_{z}\left(j\right)\left(P_{j\to i}/N_{j}\right)$$

where $N_{j}$ is the number of paths starting from the *j*-th area; and $P_{j\to i}$ is the area indicator function calculated as follows.

(4) $$P_{j\to i}=\left\{\begin{array}{c} 1\;\;\;\mathrm{when}\mathrm{there}\mathrm{is}a\mathrm{path}\mathrm{starting}\mathrm{from}\mathrm{the}j-\mathrm{th}\mathrm{area}\mathrm{to}i-\mathrm{th}\mathrm{area}\\ 0\;\;\mathrm{otherwise}\;\;\;\;\;\;\;\;\;\;\; \end{array}\right.$$

Second, by assuming that there are *g* areas in the target environment, we can obtain:

(5) $$P_{z+1}=MP_{z}$$

where $P_{z}={\left[P_{Z}\left(1\right),P_{Z}\left(2\right),...,P_{Z}\left(g\right)\right]}^{T}$; and M is a g×g matrix in which the element on the *i*-th row and *j*-th column is calculated as $m_{ij}=P_{j\to i}/N_{j}\left(1\leq i,j\leq g\right)$.

In M, $m_{ij}\left(1\leq i\leq g\right)$ becomes zero when $P_{j\to i}\left(1\leq i\leq g\right)$ is zero, which indicates that there is no path starting from the *j*-th area involved in the people’s motion patterns. In this case, we name the current area as the hung area and then distribute the same probability to all of the areas of interest at the next timestamp. On this basis, we modify the matrix M into:

(6) $$S=M+ec^{T}/g$$

where $e={\left[\underset{g\;\mathrm{in}\;\mathrm{total}}{\underset{︸}{1,1,...,1}}\right]}^{T}$; and $c={\left[c_{1},c_{2},...,c_{g}\right]}^{T}$ is an indicator vector in which each element, $c_{i}\left(1\leq \;i\leq \;g\right)$, is calculated by:

(7) $$c_{i}=\left\{\begin{array}{c} 1\;\mathrm{when}\mathrm{the}i-\mathrm{th}\mathrm{area}\mathrm{is}a\mathrm{hung}\mathrm{area}\;\;\;\;\;\;\\ 0\;\mathrm{otherwise}\;\;\;\;\;\;\;\;\;\;\; \end{array}\right.$$

After this modification, we can find that the sum of each column in S equals one, which indicates that the individual must appear in one of the areas of interest at the next timestamp. Due to the observation constraint, the area where the individual appears at the next timestamp may not be detected. To solve this problem, we set a probability factor, θ, to describe the probabilities that the individual appears nearby or in other areas at the next timestamp. Hence, we continue to modify the matrix S into:

(8) $$G=\theta S+\left(1-\theta \right)ee^{T}/g$$

**Figure 16:**
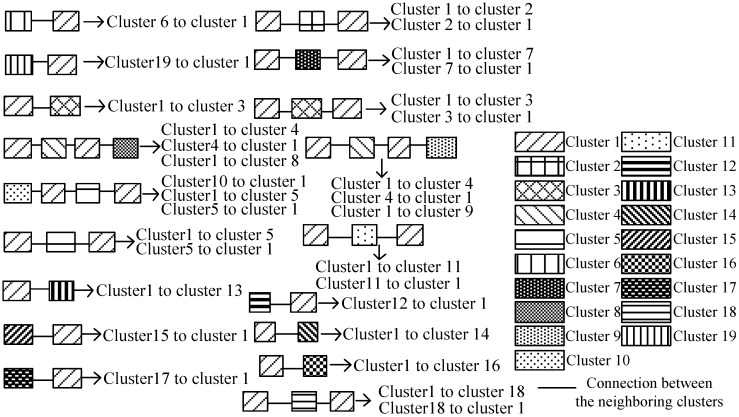
Cluster separation.

Based on Equation (8), we can find that G is a primitive matrix. Since:

(9) $$P_{z+1}=GP_{z}\to P_{z}=G^{z}P_{0},$$

we calculate the probability distribution of areas as P= lim${}_{Z\to \infty }$$P_{Z}$. By setting $P_{0}=\;{\left[\underset{g\;\mathrm{in}\;\mathrm{total}}{\underset{︸}{1/g,1/g,...,1/g}}\right]}^{T}$, which indicates that each area has the same probability to be visited at the first timestamp, we can detect the hot areas, which have been visited frequently based on the P. Similarly, the probability distribution of clusters in the signal graph can also be calculated based on the results of cluster separation in Figure 16. Table 3 shows the probabilities of the areas and clusters, respectively, in the mobility graph (MG) and the signal graph (SG), respectively.

**Table 3:** Results of probability distributions. MG, mobility graph; SG, signal graph.

IDs in MG	1	2	3	4	5	6	
Probabilities	0.27	0.11	0.05	0.22	0.03	0.09	
IDs in SG	1	2	3	4	5	6	7
Probabilities	0.19	0.03	0.026	0.03	0.026	0.01	0.02
IDs in SG	8	9	10	11	12	13	14
Probabilities	0.02	0.02	0.01	0.02	0.01	0.02	0.02
IDs in SG	15	16	17	18	19		
Probabilities	0.01	0.02	0.01	0.02	0.01		

**Figure 17:**
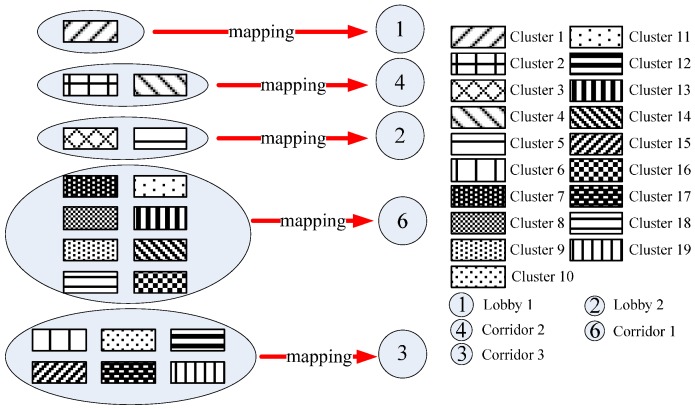
Mapping from the signal graph into the mobility graph.

Finally, we construct the mapping from the signal graph into the mobility graph based on the PR values of the areas and clusters, as shown in Figure 17. In concrete terms, we map the clusters into the areas with the same rank of PR values to preserve the consistency of the hot nodes in the mobility and signal graphs. In our experiments, Area 5 (*i.e.*, Lobby 3) cannot be mapped by any cluster, which means that this area is not a hot area, and meanwhile, there are very few RSSs collected in this area. Therefore, by using the PR algorithm, we not only construct the relation between the signal graph and the mobility graph, but also detect the hot areas that appear frequently in the people’s motion patterns.

### 3.5. Target Localization

After the mapping from the signal graph into the mobility graph is constructed, we conduct the localization by using the Kullback–Leibler (KL) divergence. Specifically, first of all, we calculate the distribution of RSS value *i* in each area, $Q_{j}^{r}\left(i\right)$, where r(1≤r≤g) is the area ID, j(1≤j≤f) is the AP ID and *f* is the AP number. Second, we calculate the KL divergence between the distribution of the newly-collected RSSs, T(i), and the RSS distribution with respect to each area, $D_{\mathrm{KL}}\left(T|\right|Q^{r})$, by:

(10) $$D_{\mathrm{KL}}\left(T|\right|Q_{j}^{r})=\prod_{j=1}^{f}(\sum_{i=1}^{\eta_{r}}T\left(i\right)\mathrm{In}\frac{T(i)}{Q_{j}^{r}\left(i\right)})$$

where $\eta_{r}$ is the maximum of RSSs.

Finally, we locate the target in the area that corresponds to the smallest KL divergence.

## 4. Performance Evaluation

### 4.1. Localization Accuracy

**Figure 18:**
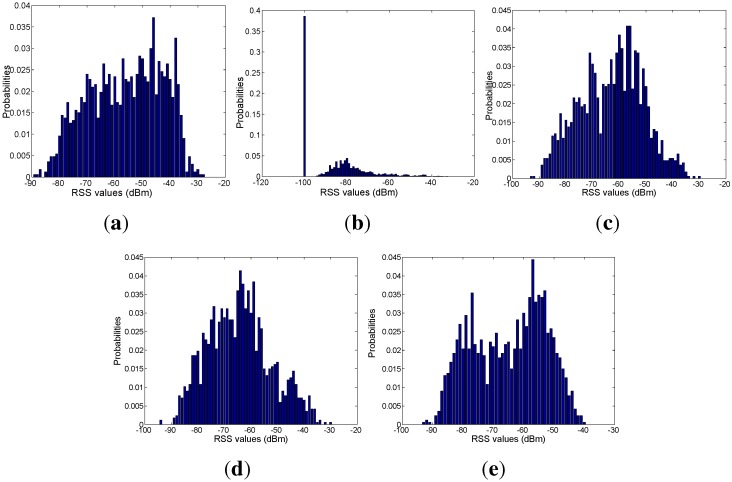
(**a**) From Access Point 1 (AP1); (**b**) from AP2; (**c**) from AP3; (**d**) from AP4; (**e**) from AP5. RSS distributions in Lobby 1.

**Figure 19:**
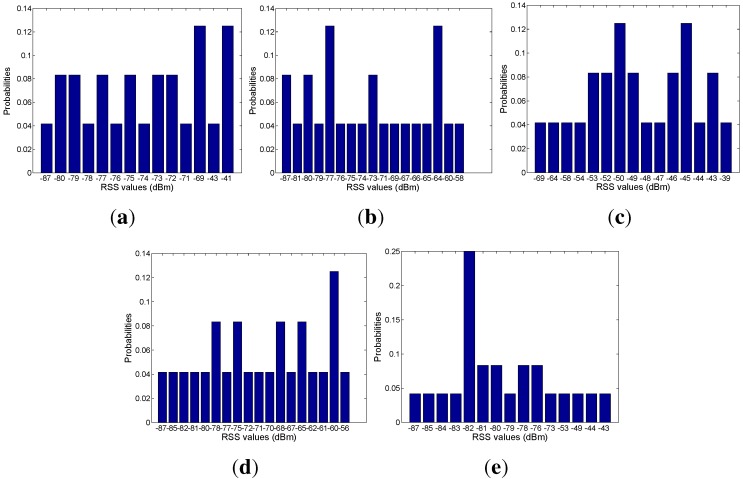
(**a**) From AP1; (**b**) from AP2; (**c**) from AP3; (**d**) from AP4; (**e**) from AP5. RSS distributions in Corridor 2.

**Figure 20:**
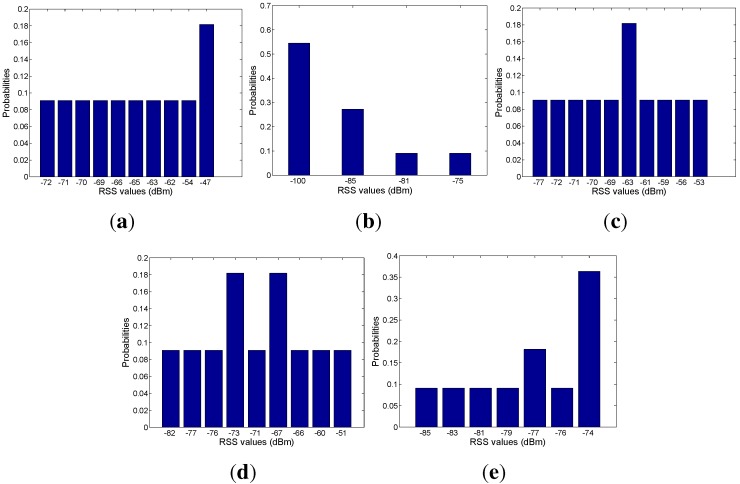
(**a**) From AP1; (**b**) from AP2; (**c**) from AP3; (**d**) from AP4; (**e**) from AP5. RSS distributions in Lobby 2.

**Figure 21:**
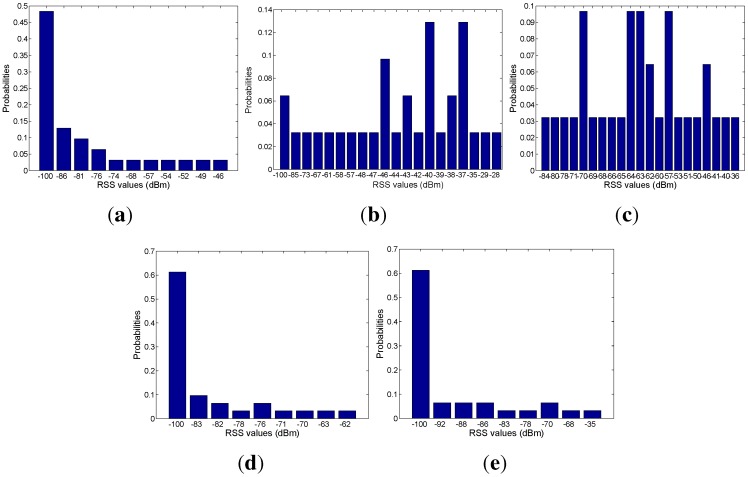
(**a**) From AP1; (**b**) From AP2; (**c**) From AP3; (**d**) From AP4; (**e**) From AP5.RSS distributions in corridor 1.

**Figure 22:**
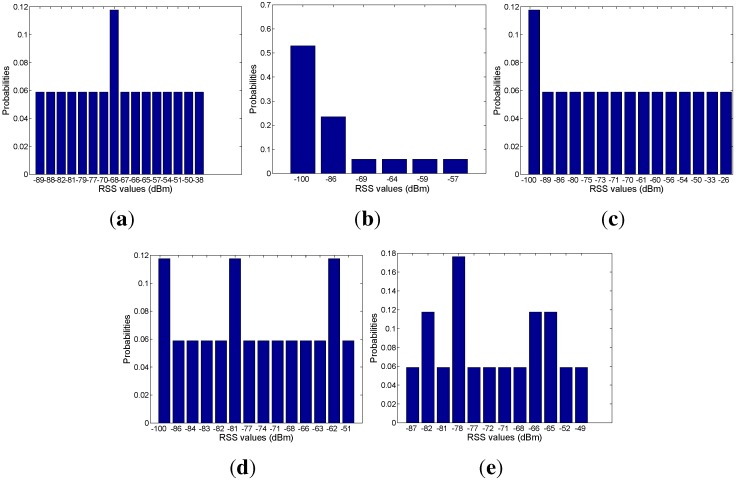
(**a**) From AP1; (**b**) from AP2; (**c**) from AP3; (**d**) from AP4; (**e**) from AP5. RSS distributions in Corridor 3.

**Figure 23:**
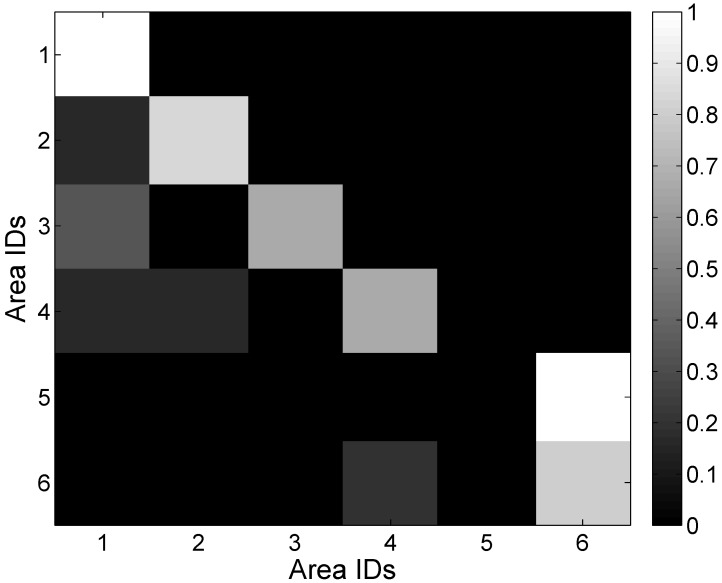
Probabilities of area localization.

Based on the mapping relation between the signal and mobility graphs, we can obtain the RSS distribution with respect to each area, as shown in Figure 18, Figure 19, Figure 20, Figure 21 and Figure 22. In our experiments, we find that the number of RSSs mapped into Lobby 1 is much larger than the number of RSSs mapped into the other areas, which means that Lobby 1 is a hot area that appears frequently in the people’s motion patterns, as expected. Figure 23 shows the result of localization accuracy. In this figure, the value on the *i*-row and *j*-th column represents the probability of the RSSs in the *i*-th area that have been estimated in the *j*-th area. As can be seen from Figure 23, the probabilities of correct area localization for Areas 1, 2, 3, 4 and 6 approach 100%, 85%, 64%, 64% and 80%, respectively. For the RSSs that have been wrongly estimated, the estimated areas are rather adjacent to the correct one, which means that the proposed approach is featured with satisfactory localization accuracy. We take Area 5 as an example. Although all of the RSSs in this area have been wrongly estimated in Area 6, Areas 5 and 6 are rather physically adjacent.

### 4.2. Parameter Discussion

**Figure 24:**
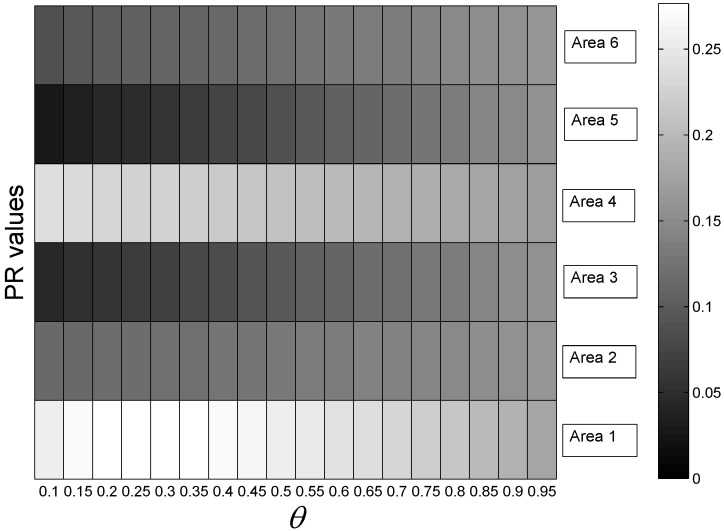
Page rank (PR) values under different values of θ for the mobility graph.

**Figure 25:**
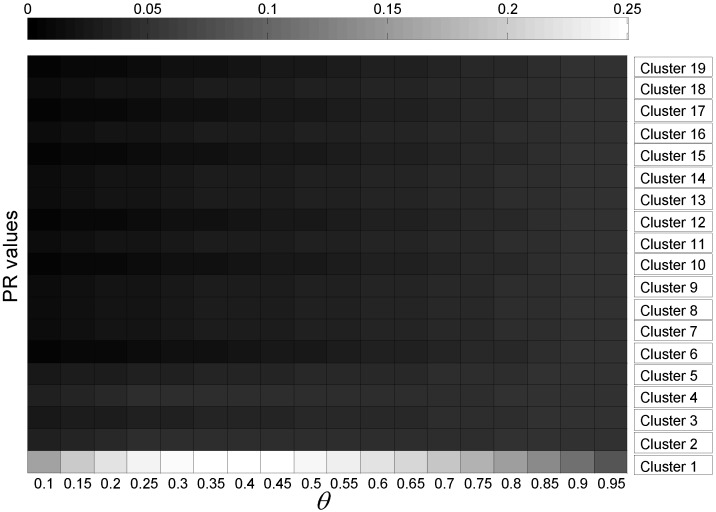
PR values under different values of θ for the signal graph.

Based on Equations (8) and (9), we can find that the value P is determined by the parameter θ. To illustrate this result more clearly, Figure 24 and Figure 25 show the variations of PR values for the mobility graph and signal graph, respectively, under different values of θ. From these figures, we observe that θ has a slight impact on the rank of PR values. Therefore, we conclude that the value of P seriously relies on the calculation of S, which means that the localization and mapping performance is significantly influenced by the observation on the motion patterns of the people in the target environment.

## 5. Conclusions and Future Work

In this paper, we propose to use a novel indoor SLAM approach, namely the PRIMAL, to characterize the layout of the target environment and consequently achieve area-level localization accuracy. Compared to the existing SLAM approaches, the PRIMAL is independent of location fingerprinting and motion sensing. In addition, the PRIMAL can not only detect the hot areas that have been visited frequently by people, but also conduct effective mapping from the signal graph into the mobility graph. Furthermore, with the help of the Allen logic, we rely on the concept of gene sequencing to investigate the correlation between different RSS sequences and meanwhile use the PR algorithm to rank the nodes in the mobility and signal graphs for better understanding of the people’s motion patterns in both the physical and signal space. For future work, how to explore the people’s motion patterns in a large-scale environment, as well as how to locate the target at the coordinate level form two interesting topics.

## Figures and Tables

**Figure A1:**
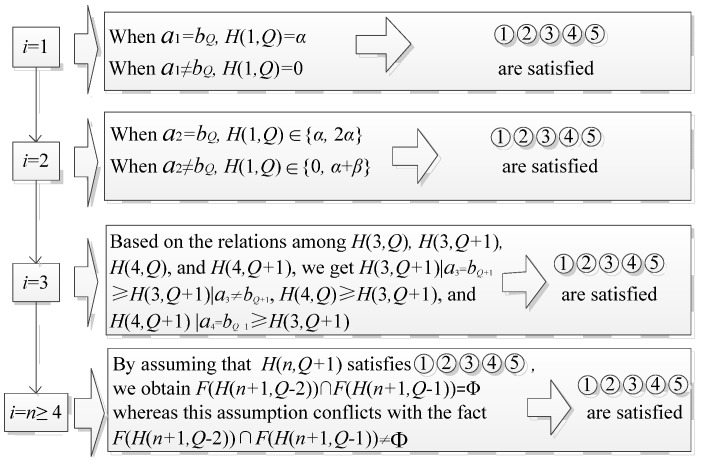
Flow chart of the proofs.

## Supplementary Materials

Supplementary materials can be accessed at: http://www.mdpi.com/1424-8220/15/10/24791/s1.

## Appendix

### Proof of the Scoring Function

The flow chart of the proofs is shown in Figure A1.

(1) Condition 1: i=1.

There are two cases to be considered as follows.

Case 1: $a_{1}=b_{1}$;

Case 2: $a_{1}\neq b_{1}$.

In Case 1, we have H(1,1)=max{0,α}=α. Then, if j=2 and $a_{1}\neq b_{2}$, we have $H\left(1,2\right)=\max\{0,\text{β},\max\left\{H\left(1-k,2\right)+w_{k}\right\},\max\left\{H\left(1,2-l\right)+w_{l}\right\}\}=0<\text{α}$; ...; if j=Q-1 and $a_{1}\neq b_{Q-1}$, we have $H\left(1,Q-1\right)=\max\{0,\text{β},\max\left\{H\left(1-k,Q-1\right)+w_{k}\right\},\max\left\{H\left(1,Q-1-l\right)+w_{l}\right\}\}=0<\text{α}$; and if j=Q and $a_{1}=b_{Q}$; we have $H\left(1,Q\right)=\max\{0,\text{α},\max\left\{H\left(1-k,Q\right)+w_{k}\right\},\max\left\{H\left(1,Q-l\right)+w_{l}\right\}\}=\text{α}>0$.

In Case 2, we have H(1,1)=max{0,β}=0. Then, if j=2 and $a_{1}\neq b_{2}$, we have H(1,2)=max{0,β}=0; ...; if j=Q-1 and $a_{1}\neq b_{Q-1}$, H(1,Q-1)=max{0,β}=0; and if j=Q and $a_{1}=b_{Q}$, $H\left(1,Q\right)=\max\{0,\text{α},\max\left\{H\left(1-k,Q\right)+w_{k}\right\},\max\left\{H\left(1,Q-l\right)+w_{l}\right\}\}=\text{α}>0$.

Therefore, we can easily obtain that H(1,j) satisfies ①②③④⑤ under Condition 1.

(2) Condition 2: i=2.

There are two cases to be considered as follows.

Case 1: $a_{2}=b_{1}$;

Case 2: $a_{2}\neq b_{1}$.

In Case 1, we have $H\left(2,1\right)=\max\{0,\text{α},\max\left\{H\left(2-k,1\right)+w_{k}\right\},\max\{H(2,1$$-l)+w_{l}\}\}=\text{α}$. Then, if j=2 and $a_{2}\neq b_{2}$, we have H(2,2)=max{0,H(1,1)$+\text{β},\max\left\{H\left(2-k,2\right)+w_{k}\right\},\max\left\{H\left(2,2-l\right)+w_{l}\right\}\}\in \{0,\text{α}+\text{β}\}<\text{α}$; ...; if j=Q-1 and $a_{2}\neq b_{Q-1}$, we have $H\left(2,Q-1\right)=\max\left\{0,H\left(1,Q-2\right)+\text{β},\max\left\{H\left(2-k,Q-1\right)+w_{k}\right\},\max\left\{H\left(2,Q-1-l\right)+w_{l}\right\}\right\}\in \{0,\text{α}+\text{β}\}<\text{α}$; and if j=Q and $a_{2}=b_{Q}$, we have $H\left(2,Q\right)=\max\left\{0,H\left(1,Q-1\right)+\text{α},\max\left\{H\left(2-k,Q\right)+w_{k}\right\},\max\left\{H\left(2,Q-l\right)+w_{l}\right\}\right\}\in \{\text{α},2\text{α}\}\geq \text{α}$.

Then, we can easily obtain that H(2,j) satisfies ①. Furthermore, the relationship between H(2,j) and H(3,j+1) is equivalent to the relationship between H(1,j-1) and H(2,j). Thus, based on the results in Condition 1, if $a_{1}=b_{j-1}$ and $a_{2}=b_{j}$, we have H(1,j-1)=α<2α=H(2,j); and if $a_{1}\neq b_{j-1}$ and $a_{2}=b_{j}$, we have H(1,j-1)=0<α=H(2,j). Hence, H(2,j) satisfies ③⑤. On the other hand, if $a_{1}=b_{j-1}$ and $a_{2}\neq b_{j}$, we have H(2,j)∈{0,α+β}<α=H(1,j-1); and if $a_{1}\neq b_{j-1}$ and $a_{2}\neq b_{j}$, we have H(2,j)=0=H(1,j-1). Hence, H(2,j) satisfies ②④.

In Case 2, we have $H\left(2,1\right)=\max\{0,\text{β},\max\left\{H\left(2-k,1\right)+w_{k}\right\},\max\{H(2,1$$-l)+w_{l}\}\}=0$. Then, if j=2 and $a_{2}\neq b_{2}$, we have H(2,2)=max{0,H(1,1)$+\text{β},\max\left\{H\left(2-k,2\right)+w_{k}\right\},\max\left\{H\left(2,2-l\right)+w_{l}\right\}\}\in \left\{0,\text{α}+\text{β}\right\}$; ...; if j=Q-1 and $a_{2}\neq b_{Q-1}$, we have $H\left(2,Q-1\right)=\max\{0,H\left(1,Q-2\right)+\text{β},\max\left\{H\left(2-k,Q-1\right)+w_{k}\right\},\max\left\{H\left(2,Q-1-l\right)+w_{l}\right\}\}\in \left\{0,\text{α}+\text{β}\right\}$; and if j=Q and $a_{2}=b_{Q}$, we have $H\left(i,j\right)=\max\{0,H\left(i-1,j-1\right)+\text{α},\max\left\{H\left(i-k,j\right)+w_{k}\right\},max\left\{H\left(i,j-l\right)+w_{l}\right\}\}\in \left\{\text{α},2\text{α}\right\}>\left\{0,\text{α}+\text{β}\right\}$.

Then, we can easily obtain that H(2,j) satisfies ①. Similarly, we equate the relationship between H(2,j) and H(3,j+1) to the relationship between H(1,j-1) and H(2,j). Based on the results in Condition 1, if $a_{1}=b_{j-1}$ and $a_{2}=b_{j}$, we have H(1,j-1)=α<2α=H(2,j); and if $a_{1}\neq b_{j-1}$ and $a_{2}=b_{j}$, we have H(1,j-1)=0<α=H(2,j). Hence, H(2,j) satisfies ③⑤. On the other hand, if $a_{1}=b_{j-1}$ and $a_{2}=b_{j}$, we have H(2,j)∈{0,α+β}<α=H(1,j-1); and if $a_{1}\neq b_{j-1}\mathrm{and}a_{2}\neq b_{j}$, we have H(2,j)≥0=H(1,j-1). Hence, H(2,j) satisfies ②④.

Therefore, we obtain that H(2,j) satisfies ①②③④⑤ under Condition 2.

In the results that follow, we only focus on the situation that j≥i in H(i,j). Similar results can be easily obtained with respect to the j<i in the H(i,j) situation.

(3) Condition 3: i=3.

In this condition, if j=3 and $a_{3}\neq b_{3}$, we have $H\left(3,3\right)=\max\left\{0,H\left(2,2\right)+\text{β},\max\left\{H\left(3-k,3\right)+w_{k}\right\},\max\left\{H\left(3,3-l\right)+w_{l}\right\}\right\}=\max\{0,H\left(2,2\right)+\text{β},H\left(2,3\right)$+β-α}∈{0,α+β,2α+β}; if j=4 and $a_{3}\neq b_{4}$, we have $H\left(3,4\right)=\max\left\{0,H\left(2,3\right)+\text{β},\max\left\{H\left(3-k,4\right)+w_{k}\right\},\max\left\{H\left(3,4-l\right)+w_{l}\right\}\right\}=\max\{0,H$(2,3)+β,H(3,3)+β-α}∈{0,α+β,2α+β,α+2β}; ...; if j=Q-1 and $a_{3}\neq b_{Q-1}$, we have $H\left(3,Q-1\right)=\max\{0,H\left(2,Q-2\right)+\text{β},\max\left\{H\left(3-k,Q-1\right)+w_{k}\right\},\max\left\{H\left(3,Q-1-l\right)+w_{l}\right\}\}\in \left\{0,\text{α}+\text{β},2\text{α}+\text{β},\text{α}+2\beta \right\}$; if j=Q and $a_{3}=b_{Q}$, we have $H\left(3,Q\right)=\max\left\{0,H\left(2,Q-1\right)+\text{α},\max\left\{H\left(3-k,Q\right)+w_{k}\right\},\max\left\{H\left(3,Q-l\right)+w_{l}\right\}\right\}=\max\left\{0,H\left(2,Q-1\right)+\text{α}\right\}\geq H\left(3,Q-1\right)$; and if j=Q+1 and $a_{3}\neq b_{Q+1}$, we have $H\left(3,Q+1\right)=\max\{0,H\left(2,Q\right)+\text{β},\max\left\{H\left(3-k,Q+1\right)+w_{k}\right\},\max\left\{H\left(3,Q+1-l\right)+w_{l}\right\}\}$. To obtain the relationship between H(3,Q) and H(3,Q+1), we need to examine the relations of four neighboring elements, H(3,Q), H(3,Q+1), H(4,Q) and H(4,Q+1). If $a_{4}=b_{Q}$, the calculation of H(4,Q) is equivalent to the calculation of H(3,Q+1) with $a_{3}=b_{Q+1}$. Since $H\left(3,Q+1\right){|}_{a_{3}=b_{Q+1}}>H\left(3,Q+1\right){|}_{a_{3}\neq b_{Q+1}}$, we have H(4,Q)≥H(3,Q+1); and if $a_{4}=b_{Q+1}$, we have $H\left(4,Q+1\right)=\max\left\{0,H\left(3,Q\right)+\text{α},\max\left\{H\left(4-k,Q+1\right)+w_{k}\right\},\max\left\{H\left(4,Q+1-l\right)+w_{l}\right\}\right\}=\max\left\{0,2\text{α}+H\left(2,Q-1\right),\max\left\{H\left(4-k,Q+1\right)+w_{k}\right\},\max\left\{H\left(4,Q+1-l\right)+w_{l}\right\}\right\}\geq 2\text{α}+\text{β}\geq H\left(3,Q+1\right)$.

Therefore, we can easily obtain that H(3,j) satisfies ①②③④⑤ under Condition 3.

(4) Condition 4: i=n≥4.

In this condition, if j=n and $a_{n}\neq b_{n}$, we have $H\left(n,n\right)=\max\left\{0,H\left(n-1,n-1\right)+\text{β},\max\left\{H\left(n-k,n\right)+w_{k}\right\},\max\left\{H\left(n,n-l\right)+w_{l}\right\}\right\}\in \left\{0,\text{α}+\left(n-1\right)\text{β},\text{α}+\left(n-2\right)\text{β},...,\text{α}+\text{β},2\text{α}+\left(n-2\right)\text{β},...,2\text{α}+\text{β},...\left(n-1\right)\text{α}+\text{β}\right\}$; ...; if j=Q-1 and $a_{n}\neq b_{Q-1}$, we have $H\left(n,Q-1\right)=\max\left\{0,H\left(n-1,Q-2\right)+\text{β},\max\left\{H\left(n-k,Q-1\right)+w_{k}\right\},\max\left\{H\left(n,Q-1-l\right)+w_{l}\right\}\right\}\in \left\{0,\text{α}+\left(n-1\right)\text{β},\text{α}+\left(n-2\right)\text{β},...,\text{α}+\text{β},2\text{α}+\left(n-2\right)\text{β},...,2\text{α}+\text{β},...\left(n-1\right)\text{α}+\text{β}\right\}$; if j=Q and $a_{n}=b_{Q}$, we have $H\left(n,Q\right)=\max\{0,H\left(n-1,Q-1\right)+\text{α},\max\left\{H\left(n-k,Q\right)+w_{k}\right\},\max\left\{H\left(n,Q-l\right)+w_{l}\right\}\}$; and if j=Q+1 and $a_{n}\neq b_{Q+1}$, we assume that H(n,Q+1) satisfies ①②③④⑤.

Then, under the $a_{n+1}\neq b_{Q-1}$ and $a_{n+1}=b_{Q}$ conditions, if H(n+1,Q)<H(n+1,Q-1), we obtain:

(11) $$\max\left\{\begin{array}{c} 0\\ \{H(n+1,Q-1)-\\ \text{α}+\text{β}\}\\ H(n,Q-1)+\text{α}\\ H(n,Q)-\text{α}+\text{β} \end{array}\right\}<\max\left\{\begin{array}{c} 0\\ \{H(n+1,Q-2)-\\ \text{α}+\text{β}\}\\ H(n,Q-1)+\text{β}\\ H(n,Q-1)-\text{α}+\text{β} \end{array}\right\}$$

Based on Equation (11), since under the $a_{n}\neq b_{Q-2},a_{n}=b_{Q-1}$ and $a_{n}\neq b_{Q}$ conditions, we have H(n,Q-1)≥H(n,Q-2) and H(n,Q-1)≥H(n,Q), Equation (11) can be simplified into H(n+1,Q-2)>H(n+1,Q-1). Thus, the range of the value H(n+1,Q-2), F(H(n+1,Q-2)), is not overlapped with the range of the value H(n+1,Q-1), F(H(n+1,Q-1)), notated as F(H(n+1,Q-2))∩F(H(n+1,Q-1))=Φ.

On the other hand, the relations of H(n+1,Q-2)∈{0,α,α+β,...,α+nβ,2α,2α+β,...,2α+(n-1)β,nα,nα+β,(n+1)α} and H(n+1,Q-1)∈{0,α+β,...,α+nβ,2α+β,...,2α+(n-1)β,nα+β} indicate that F(H(n+1,Q-2))∩F(H(n+1,Q-1))≠Φ, which conflicts with the previous assumption in (Equation (11)). Therefore, we obtain that H(n+1,Q)≥H(n+1,Q-1), and thereby, H(n+1,Q) satisfies ①②③④⑤ under Condition 4.

In conclusion, it is proven that the definition of H(i,j) satisfies ①②③④⑤.
