# Supplementary material for: PRIMAL: Page Rank-Based Indoor Mapping and Localization Using Gene-Sequenced Unlabeled WLAN Received Signal Strength
Source: Sensors (Basel). 2015 Sep 25;15(10):24791–817. doi: 10.3390/s151024791 (PMC4634416; doi:10.3390/s151024791)
Supplement: Supplementary file 1 [file sensors-15-24791-s001.pdf]

OPEN ACCESS

sensors

ISSN 1424-8220

www.mdpi.com/journal/sensors

Supplementary Information

PRIMAL: Page Rank-Based Indoor Mapping and Localization Using Gene-Sequenced Unlabeled WLAN Received Signal Strength. *Sensors* 2015, *15*, 24791-24817

Mu Zhou [1,*], Qiao Zhang [1], Kunjie Xu [2], Zengshan Tian [1], Yanmeng Wang [1] and Wei He [1]

[1] Chongqing Key Lab of Mobile Communications Technology, Chongqing University of Posts and Telecommunications, Chongqing 400065, China; E-Mails: zhangqiao6120@gmail.com (Q.Z.); tianzs@cqupt.edu.cn (Z.T.); 2012210036@stu.cqupt.edu.cn (Y.W.); hewei@cqupt.edu.cn (W.H.)

[2] Ericsson, San Jose, CA 95134, USA; E-Mail: xu.kunjie@gmail.com

* Author to whom correspondence should be addressed; E-Mail: zhoumu@cqupt.edu.cn; Tel.: +86-139-8385-0201; Fax: +86-23-6248-7993.

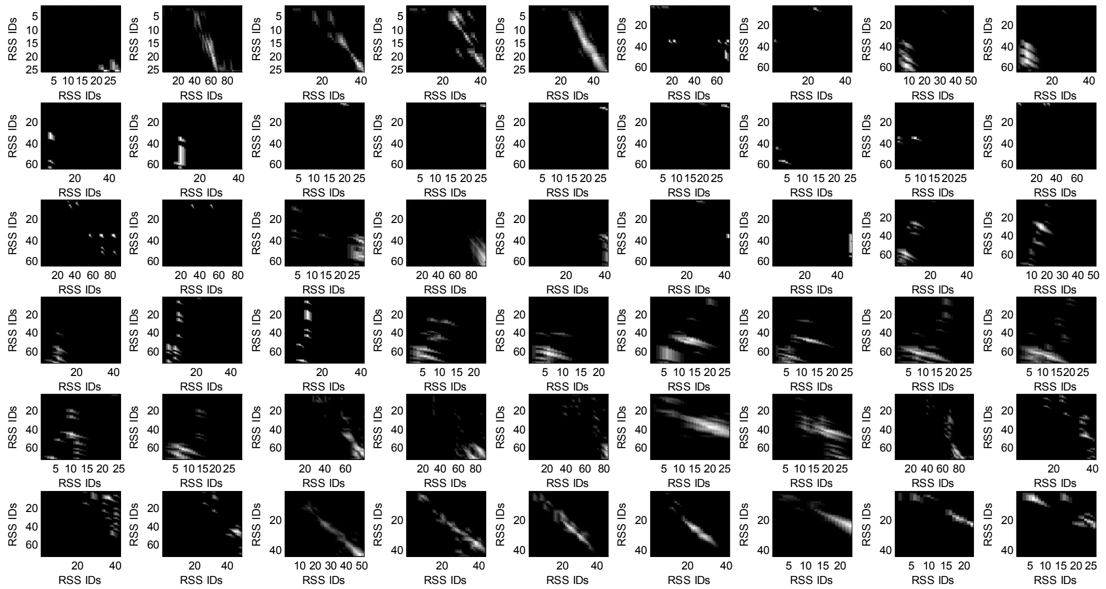

S2

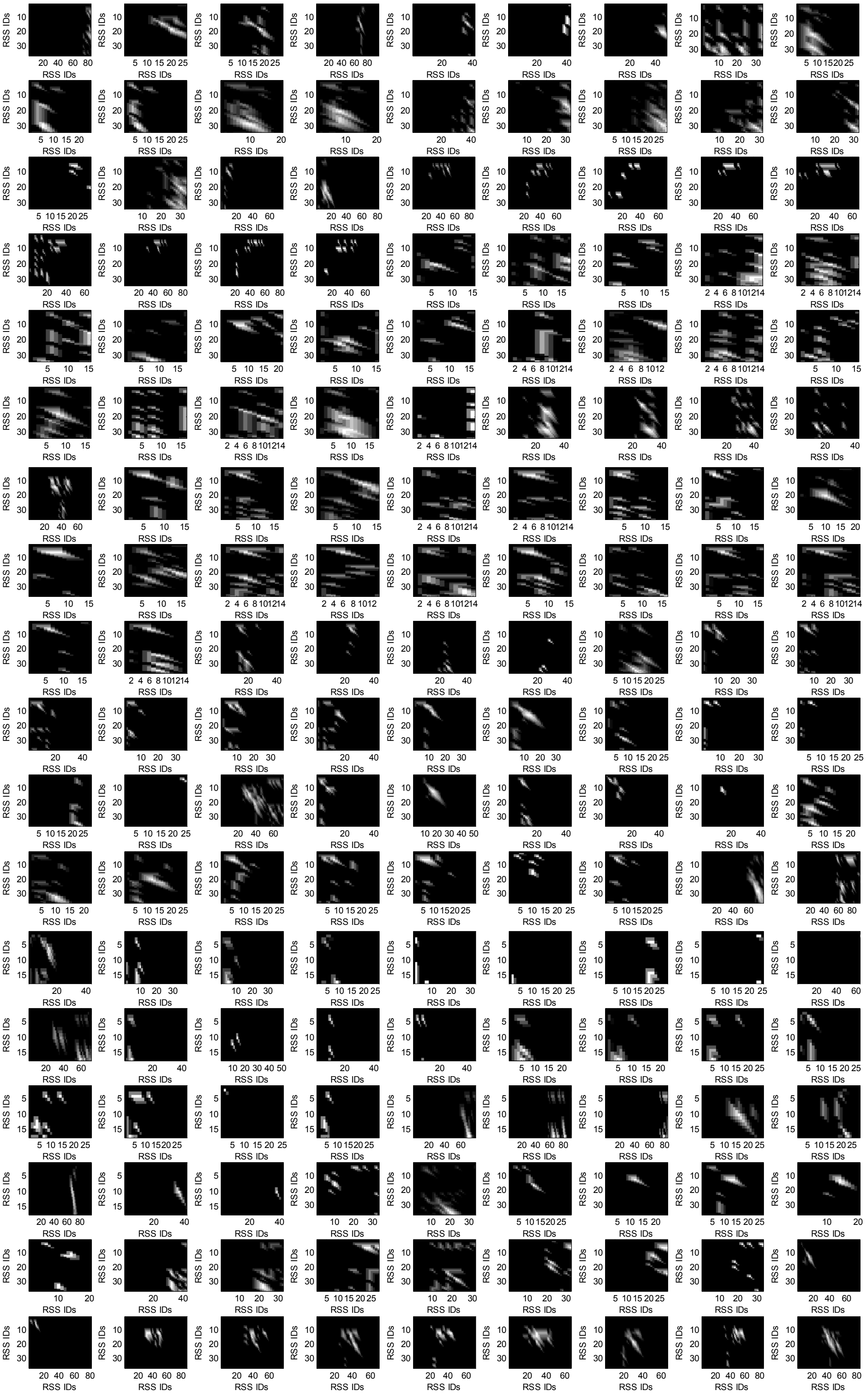

S3

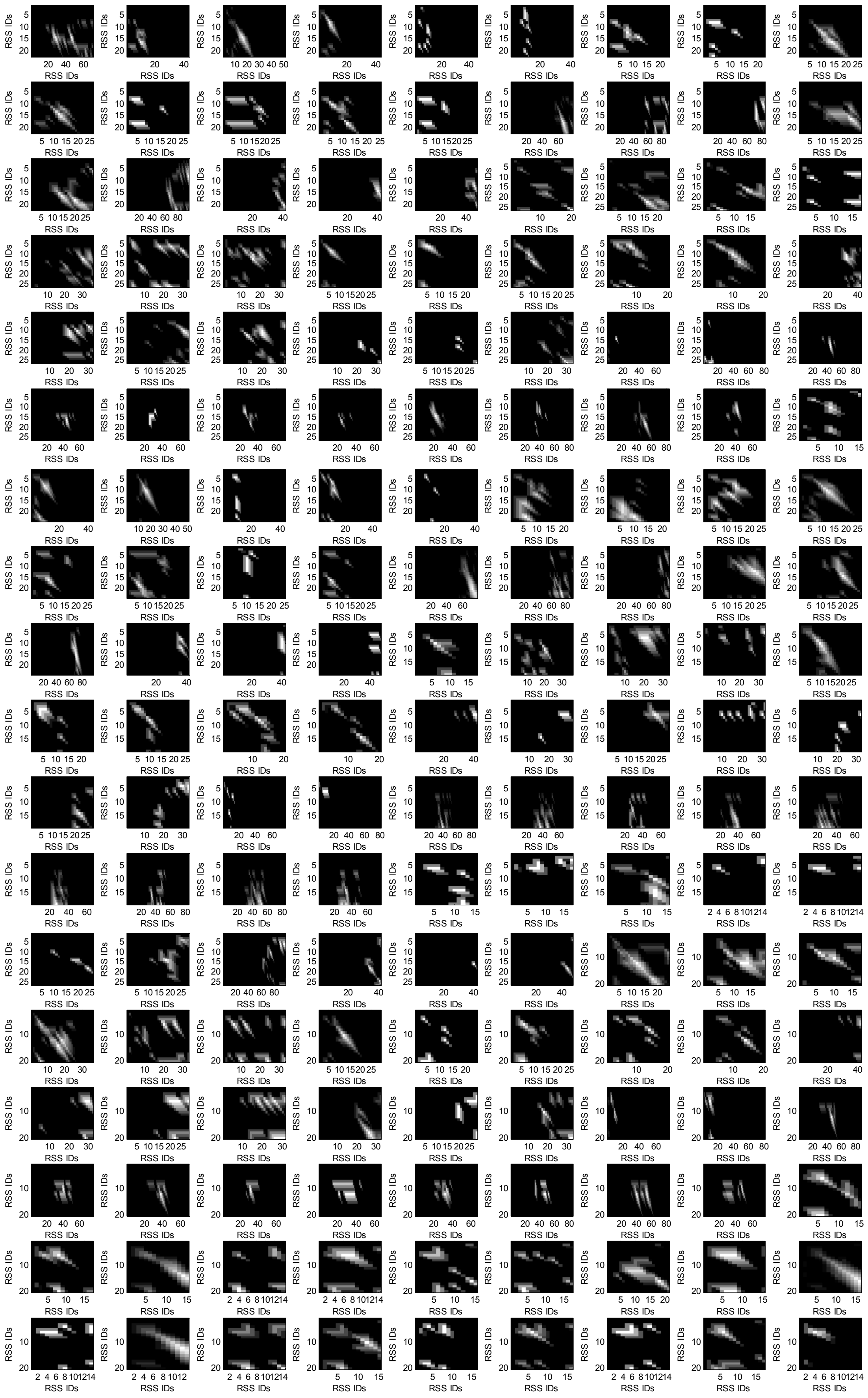

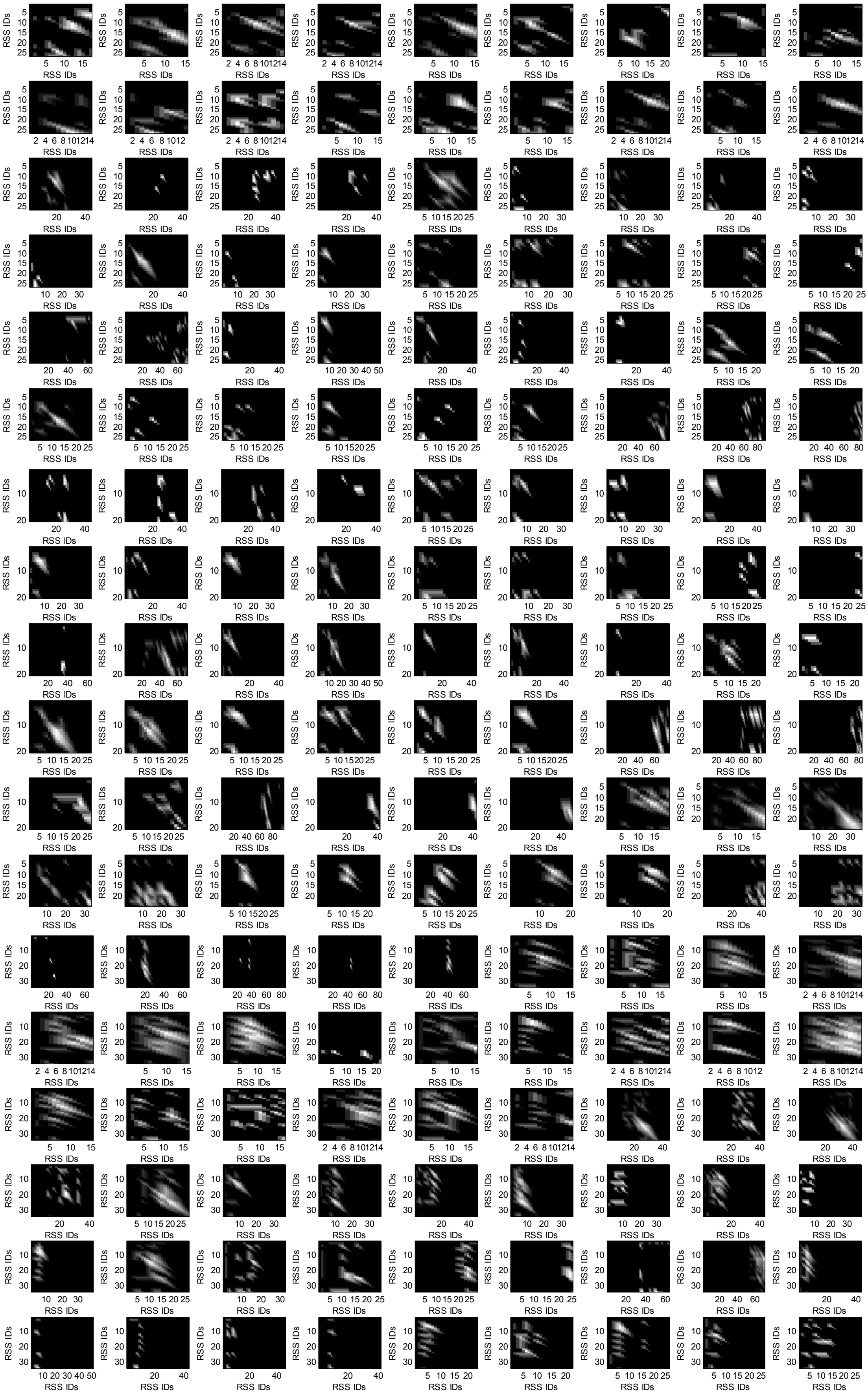
RSS IDs

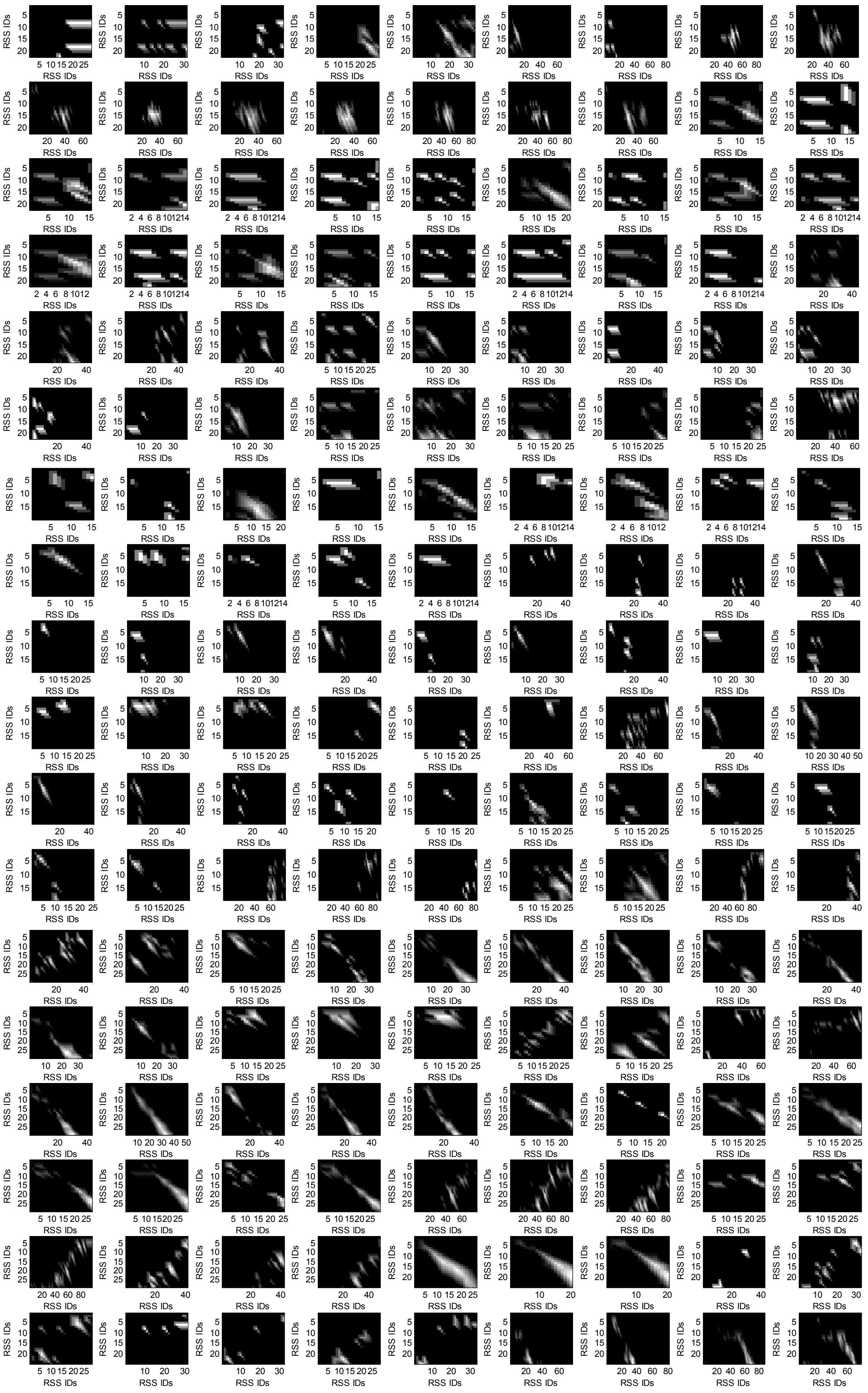
RSS IDs

S6

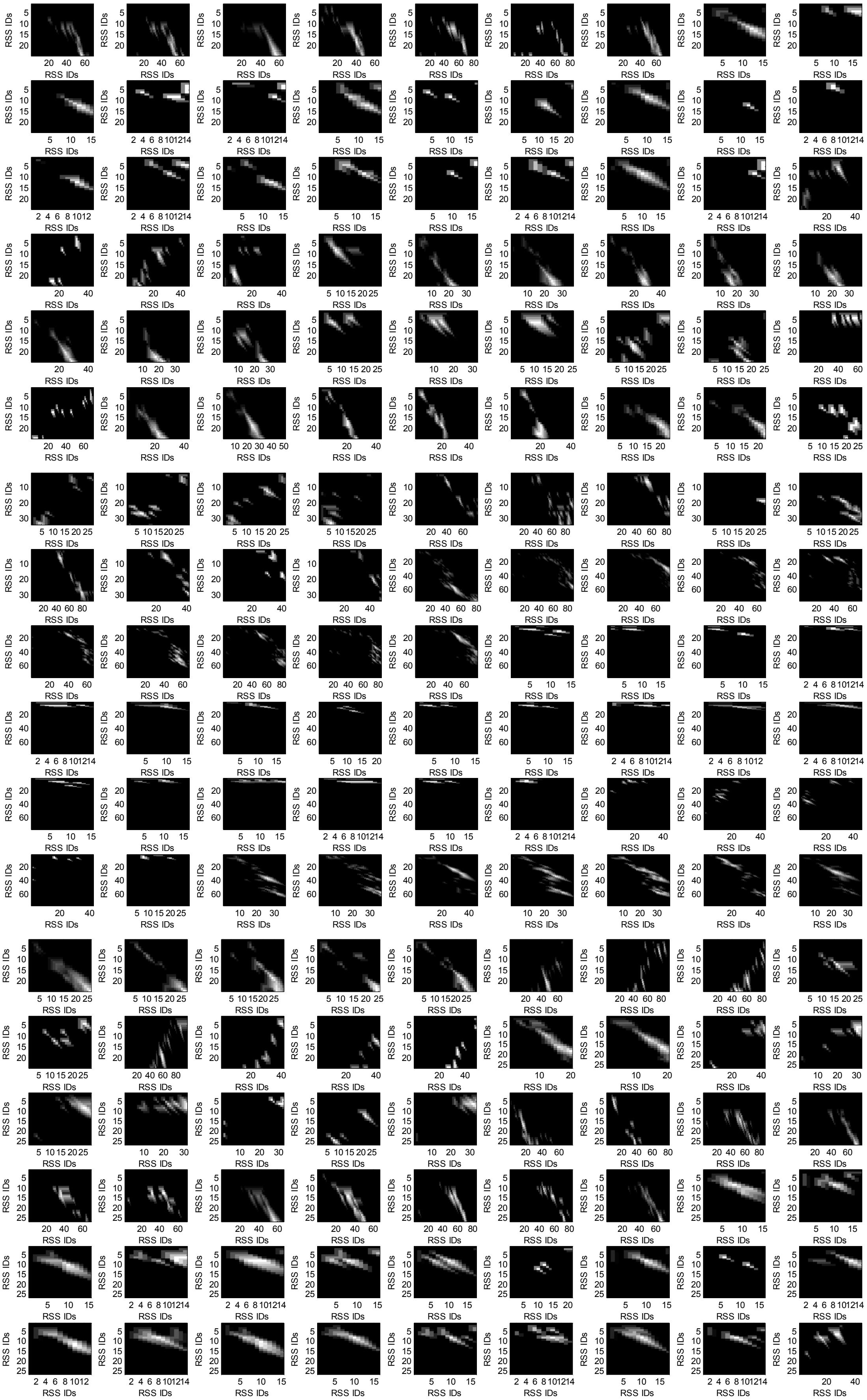

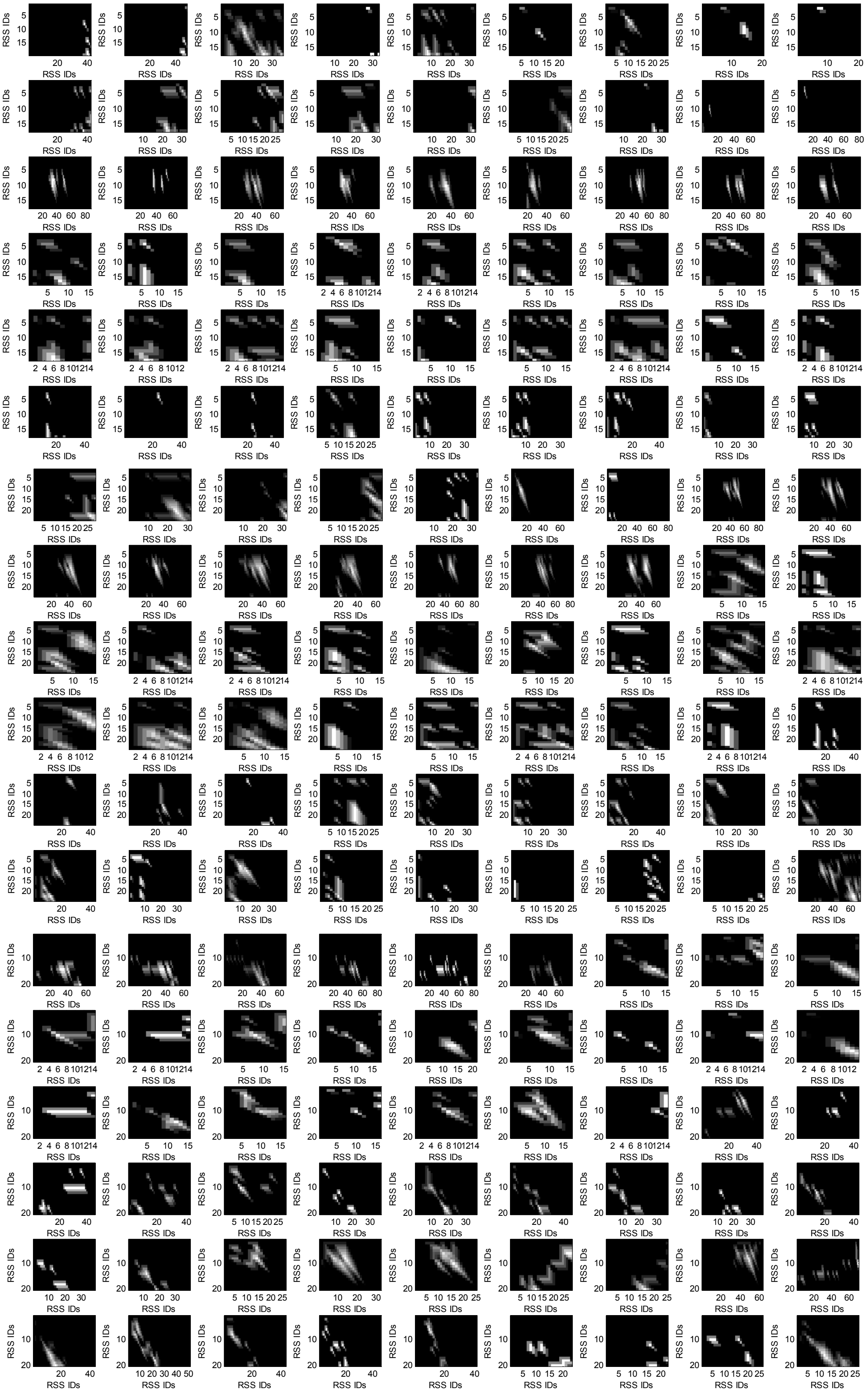
RSS IDs

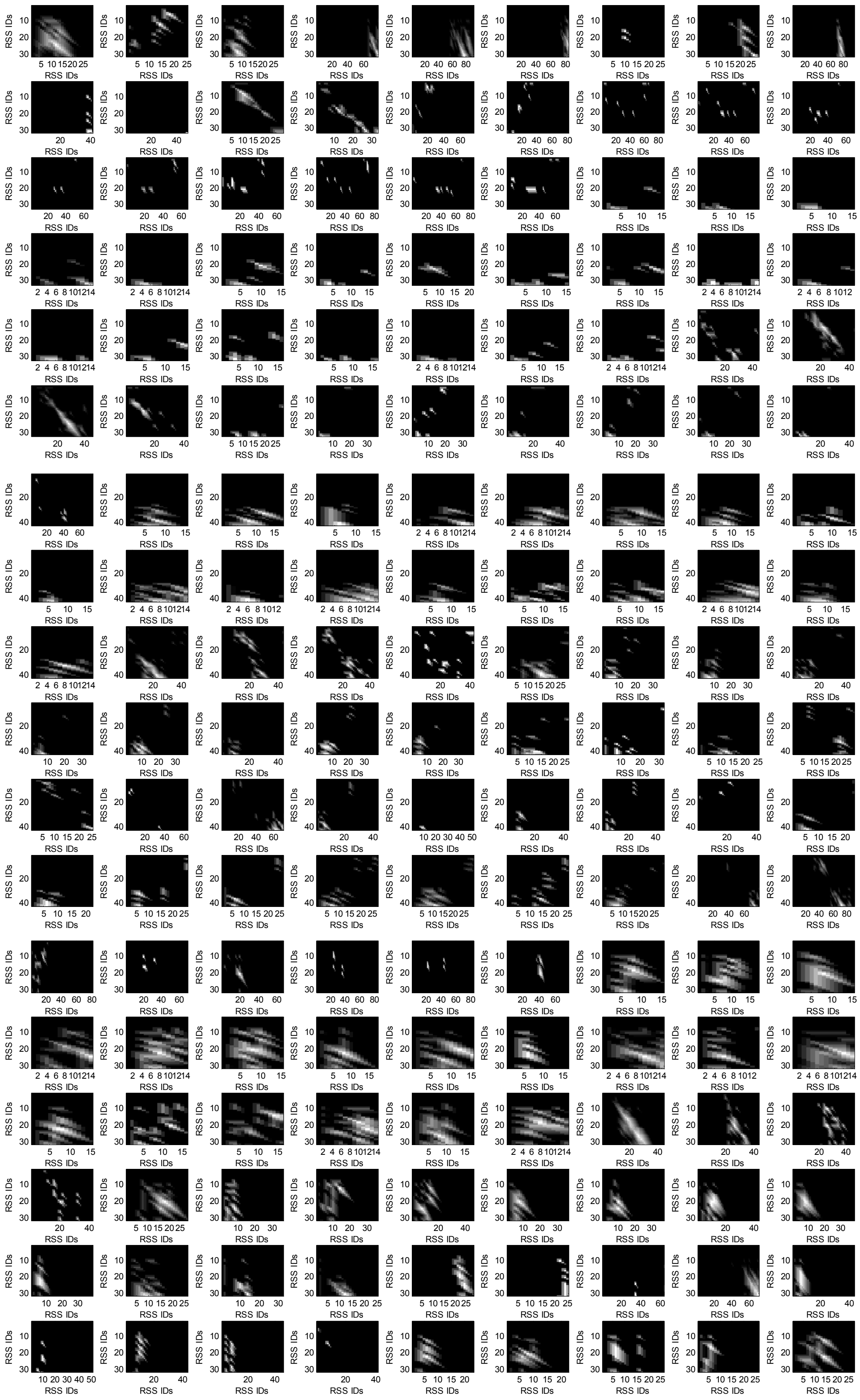
RSS IDs

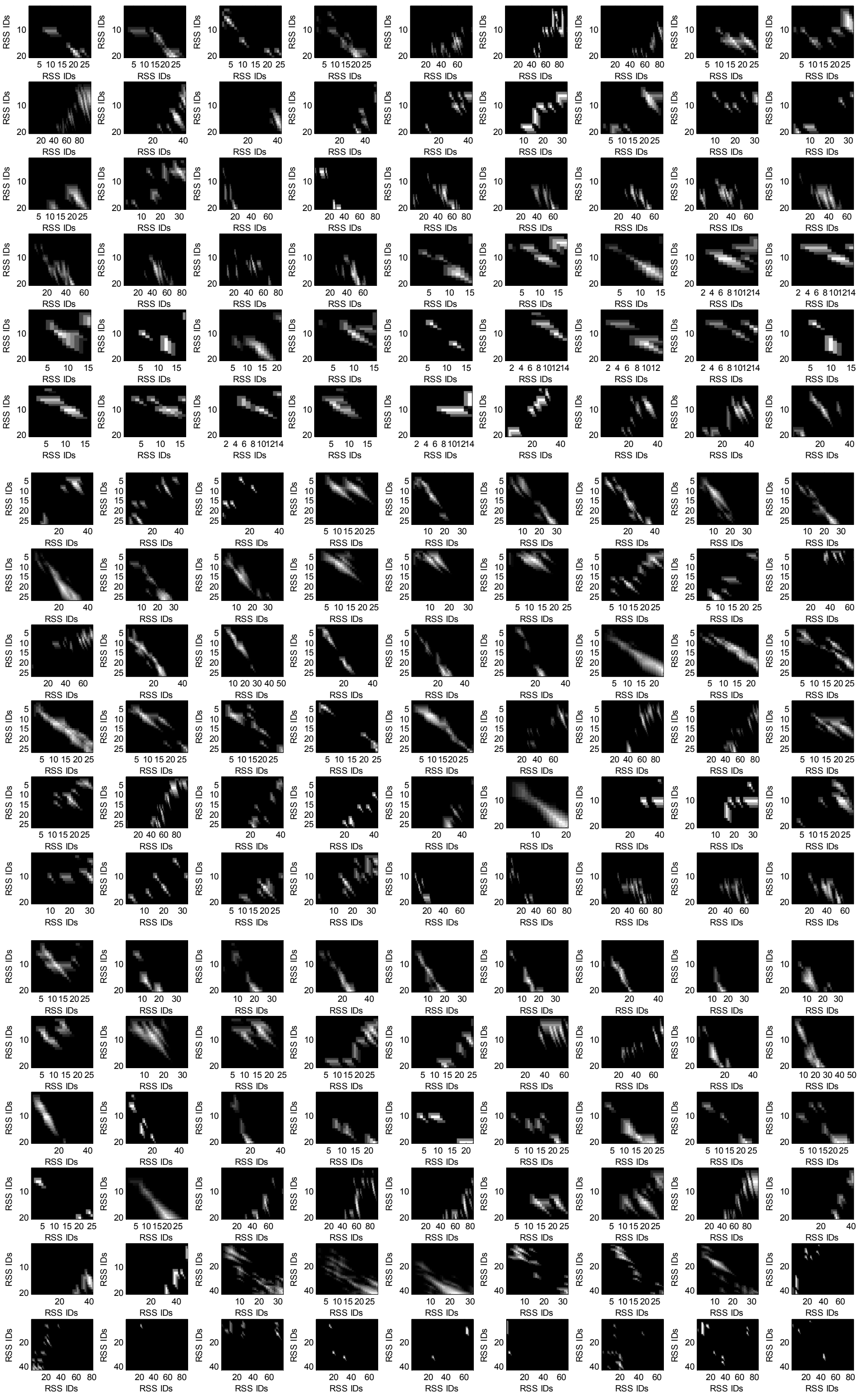
RSS IDs

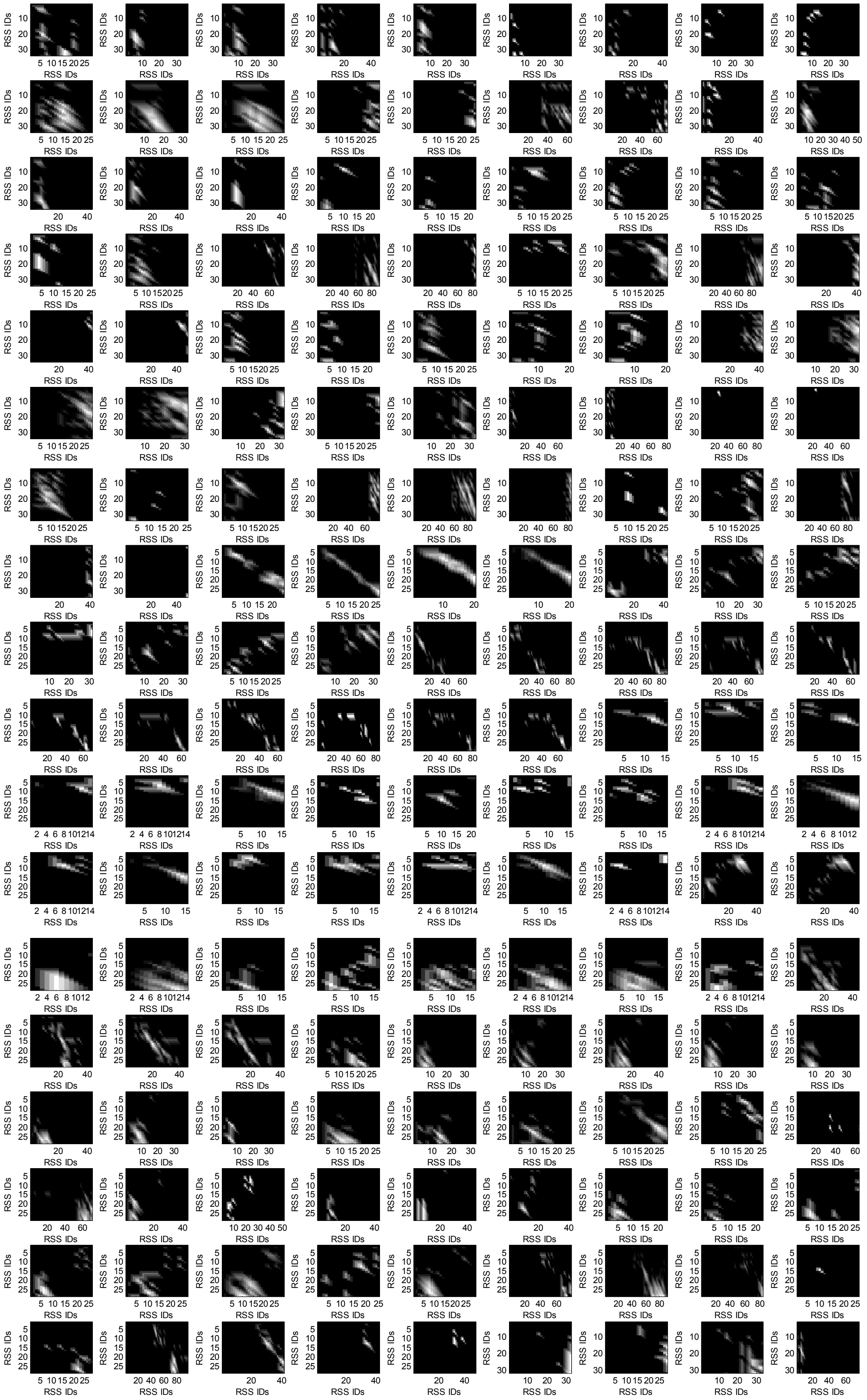
RSS IDs

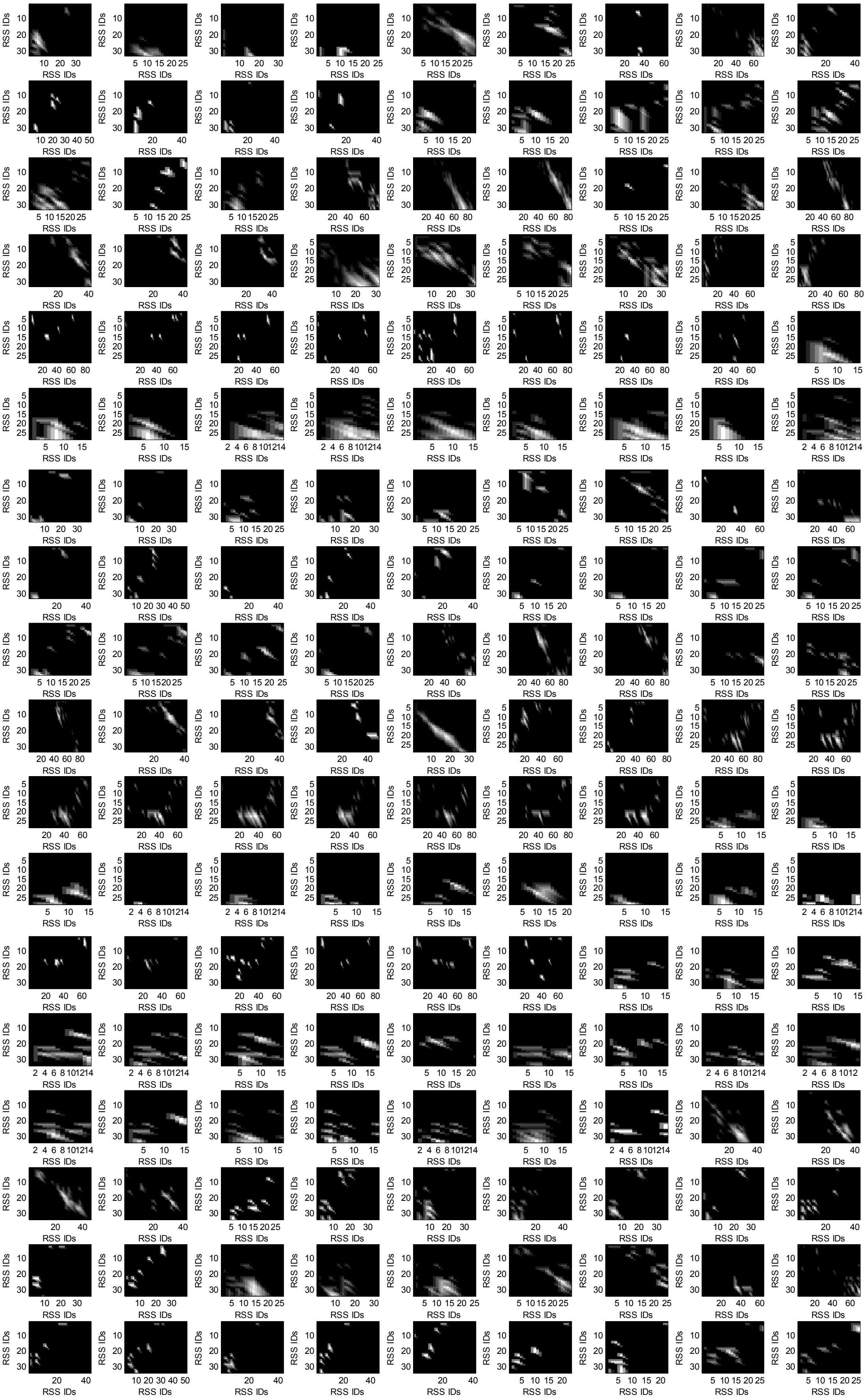
RSS IDs

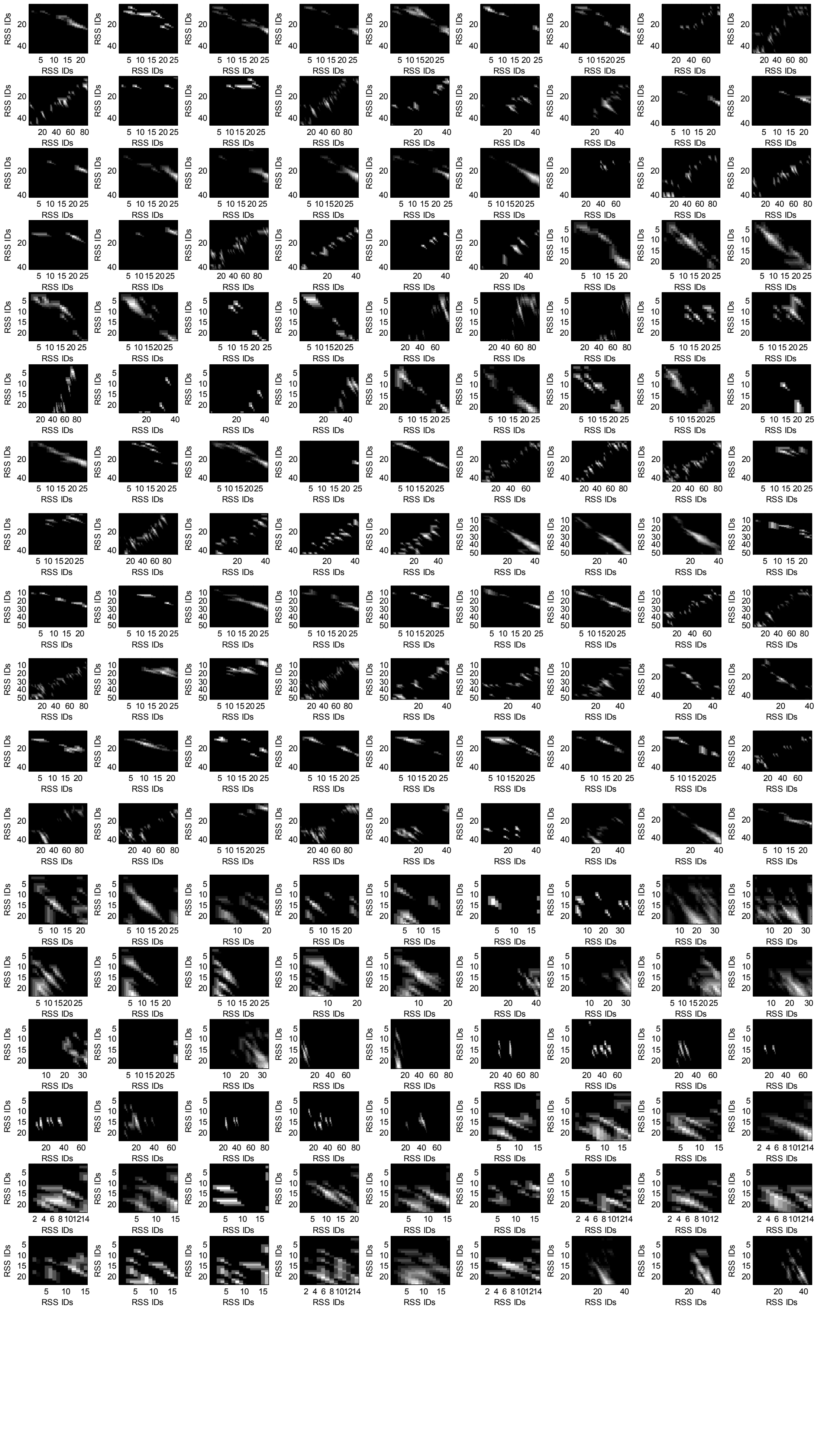
RSS IDs

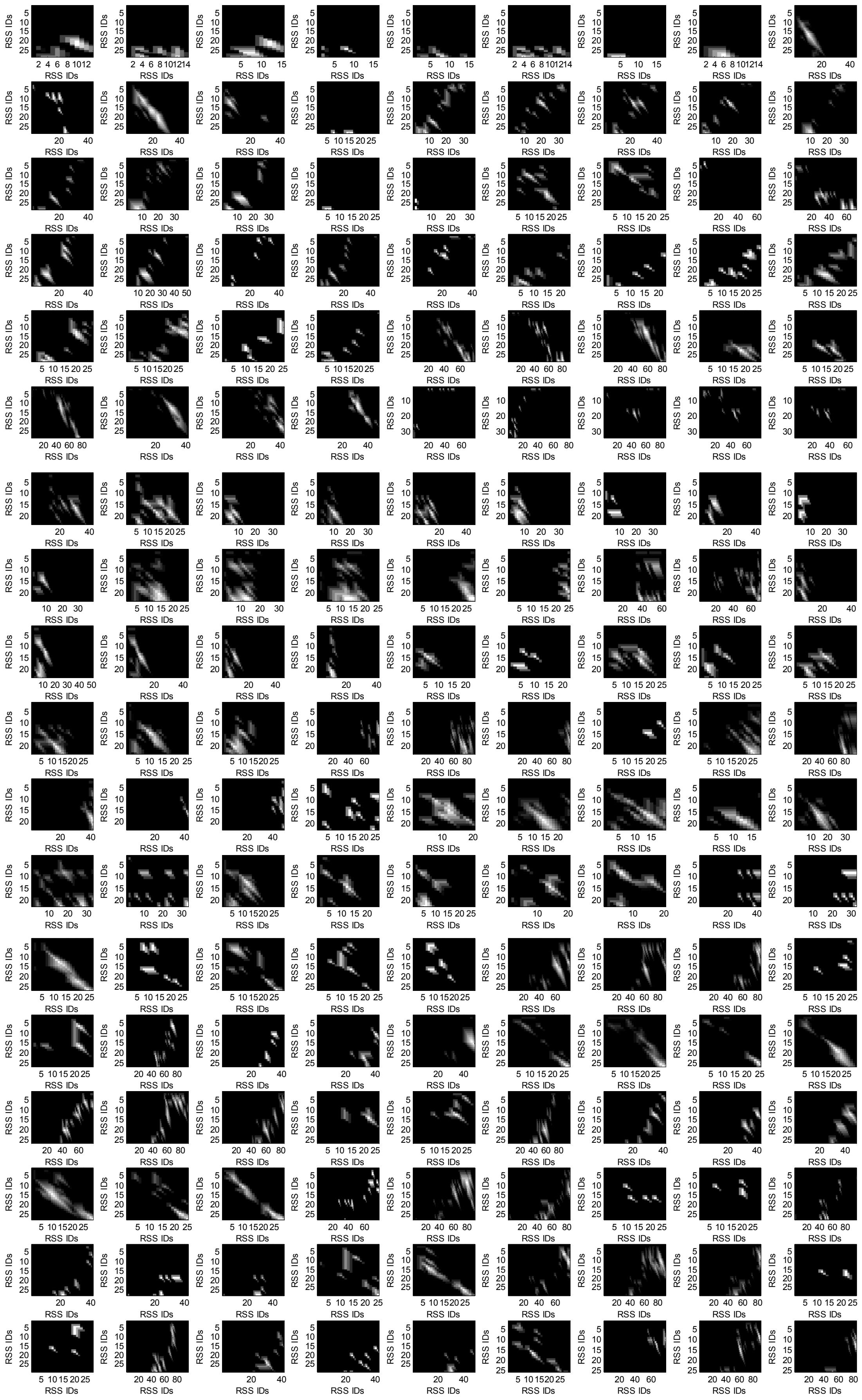
RSS IDs

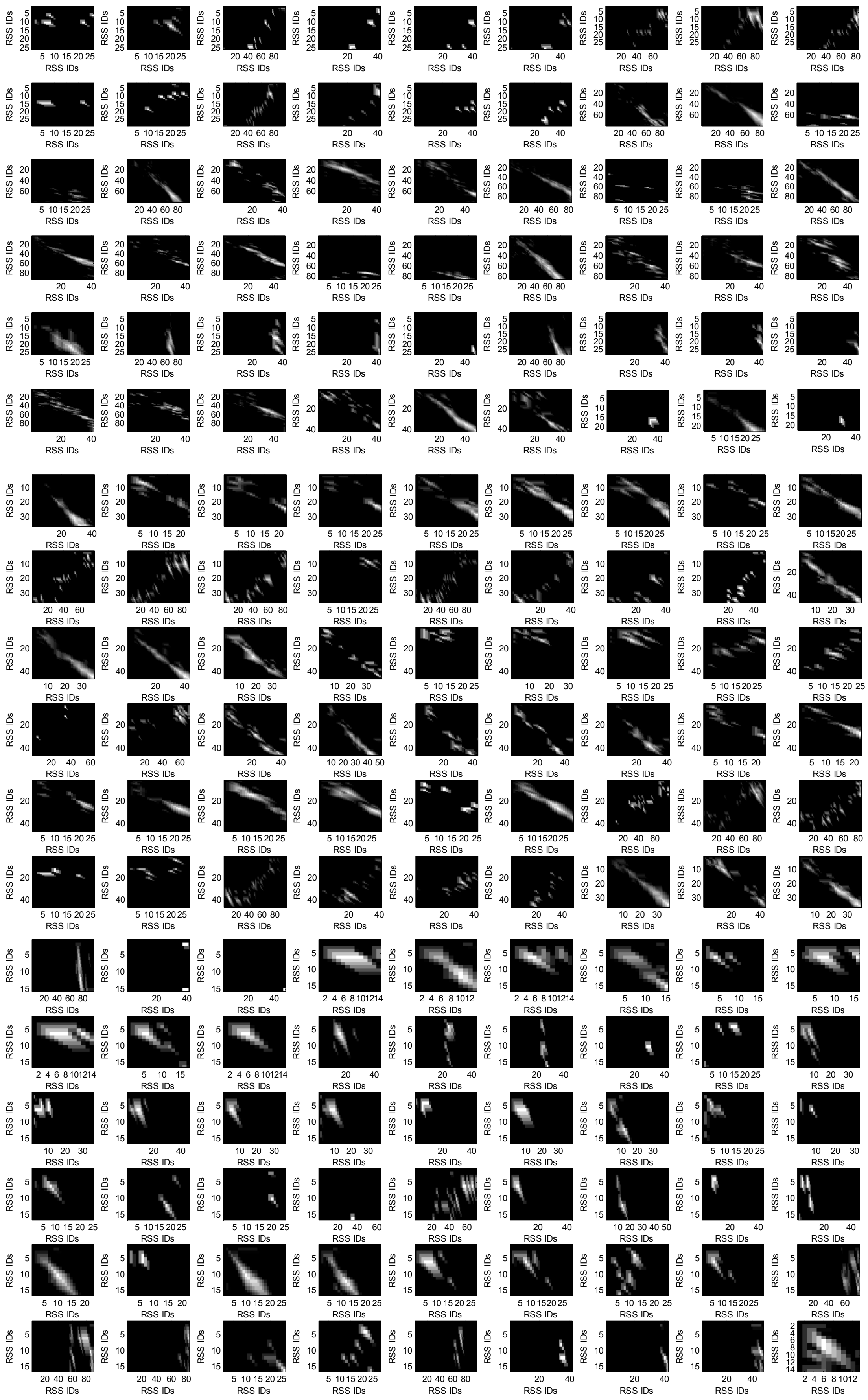
RSS IDs

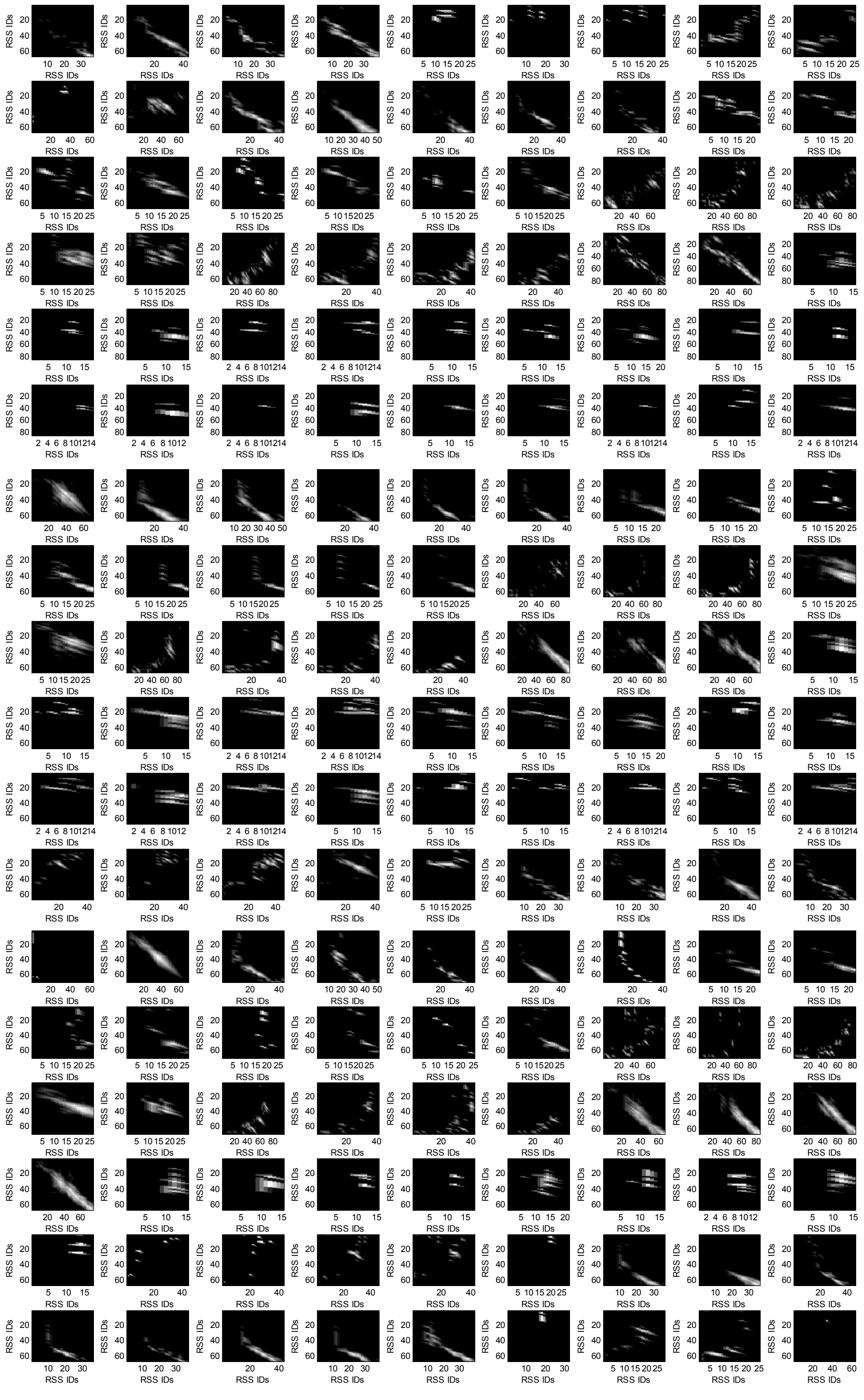
RSS IDs
RSS IDs

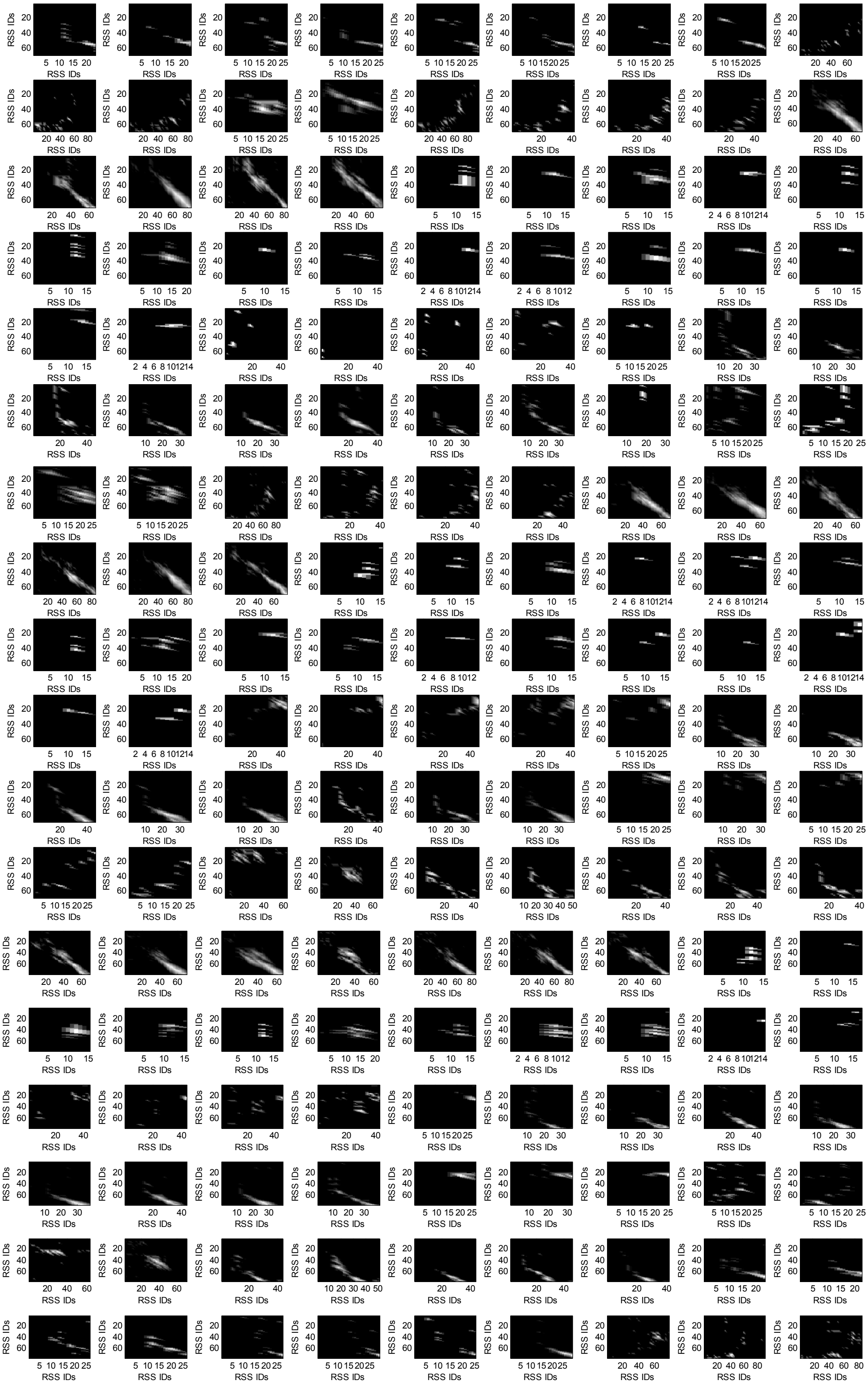
RSS IDs

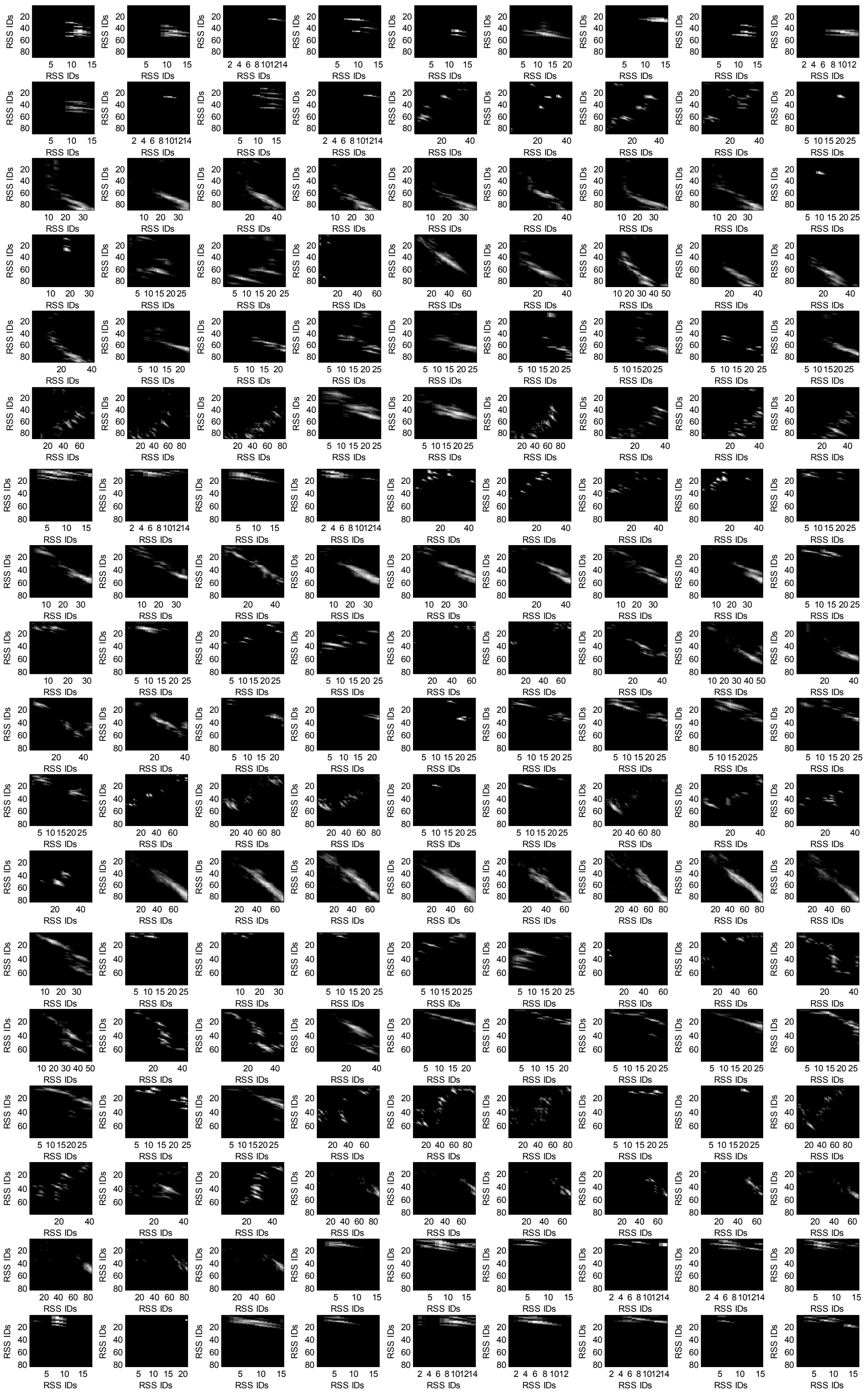
RSS IDs

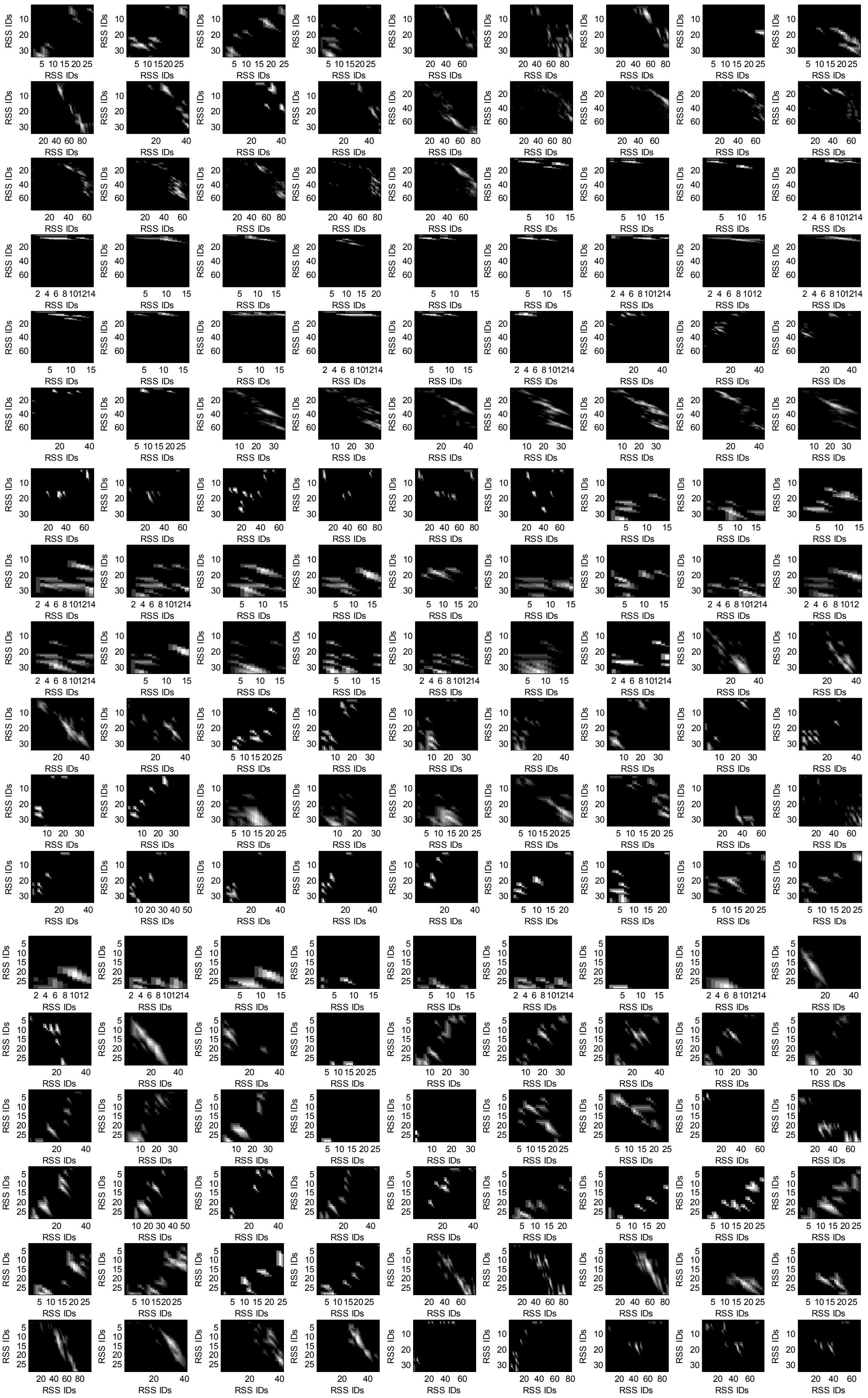
RSS IDs

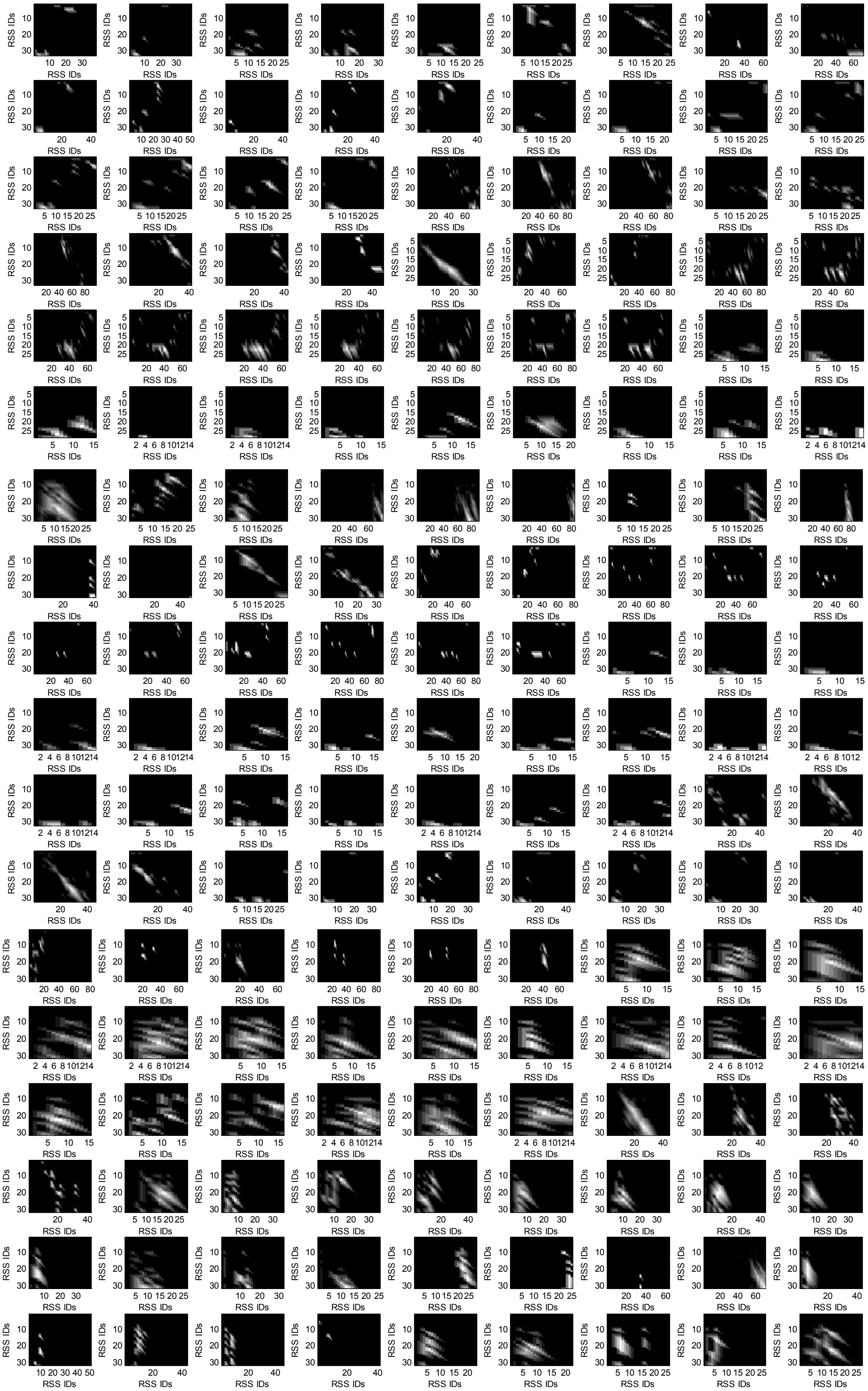
RSS IDs

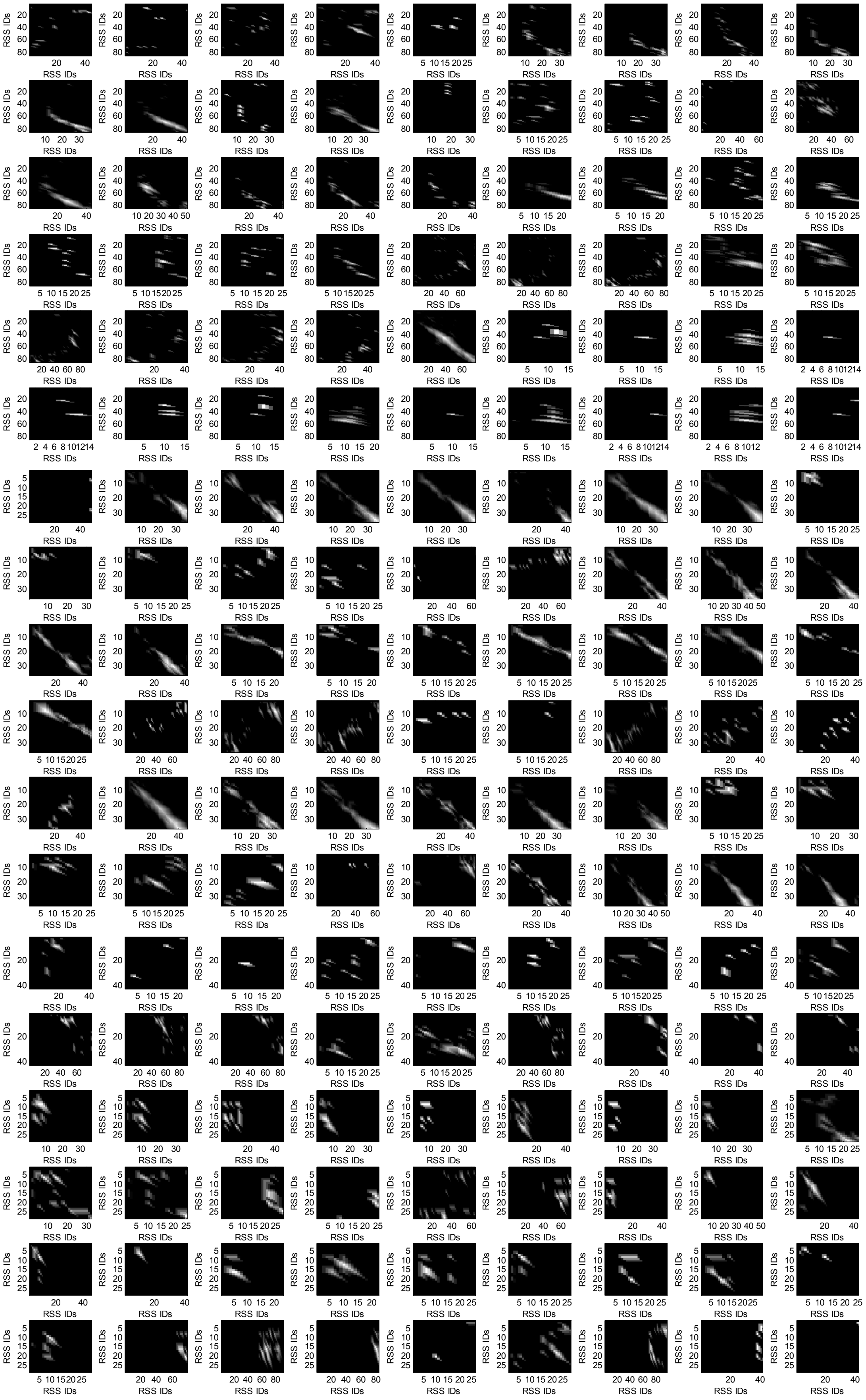
RSS IDs

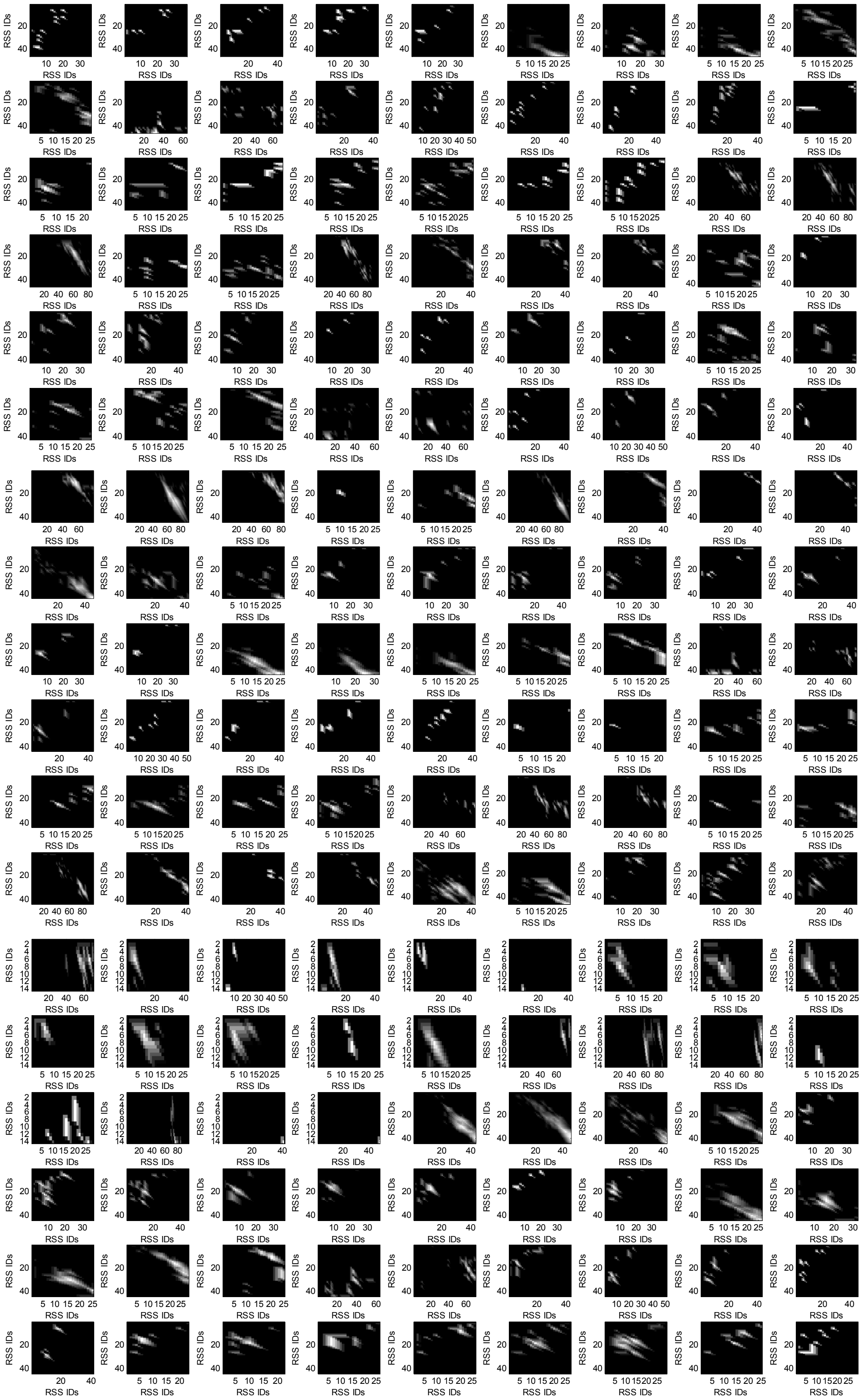
RSS IDs

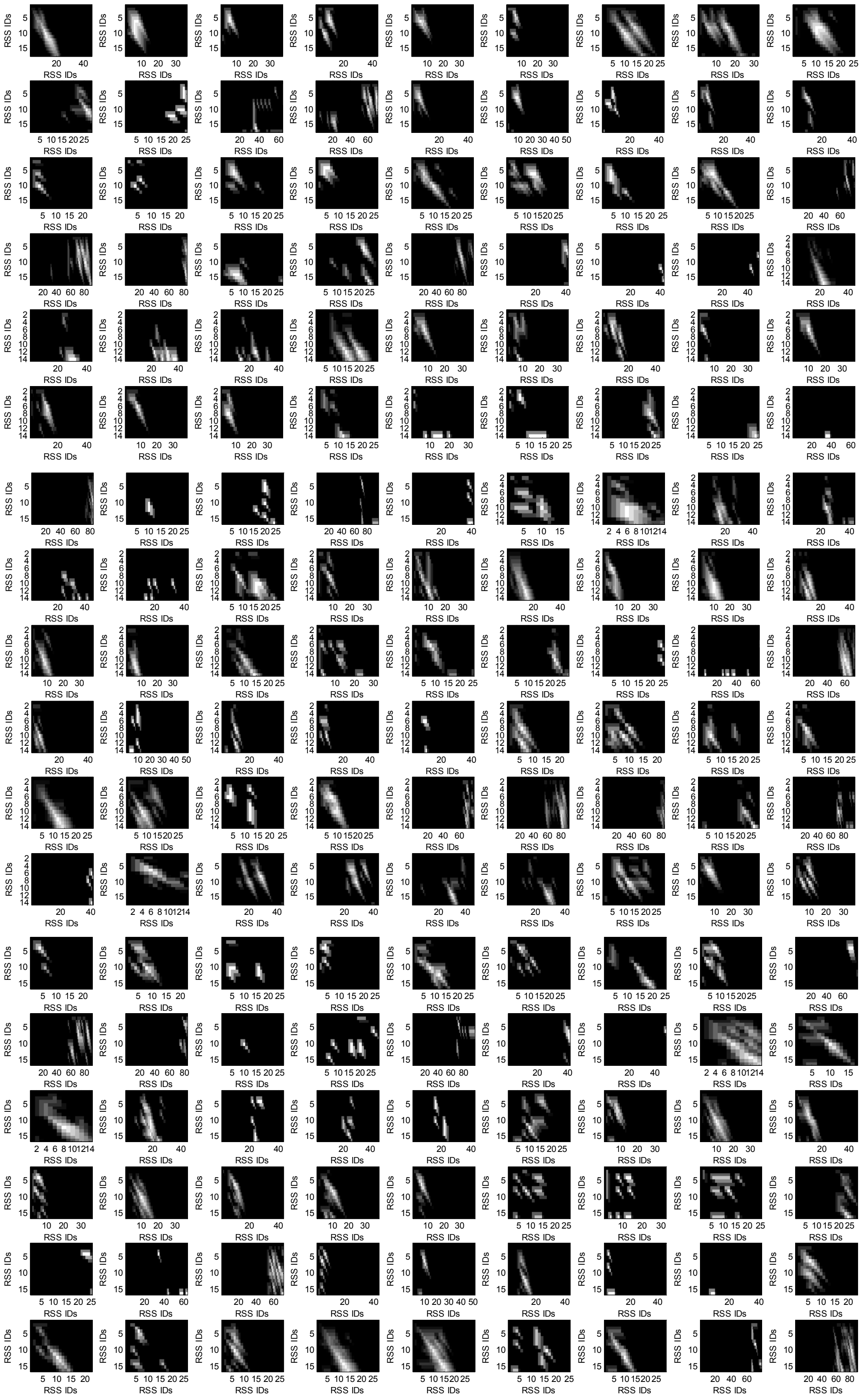
RSS IDs

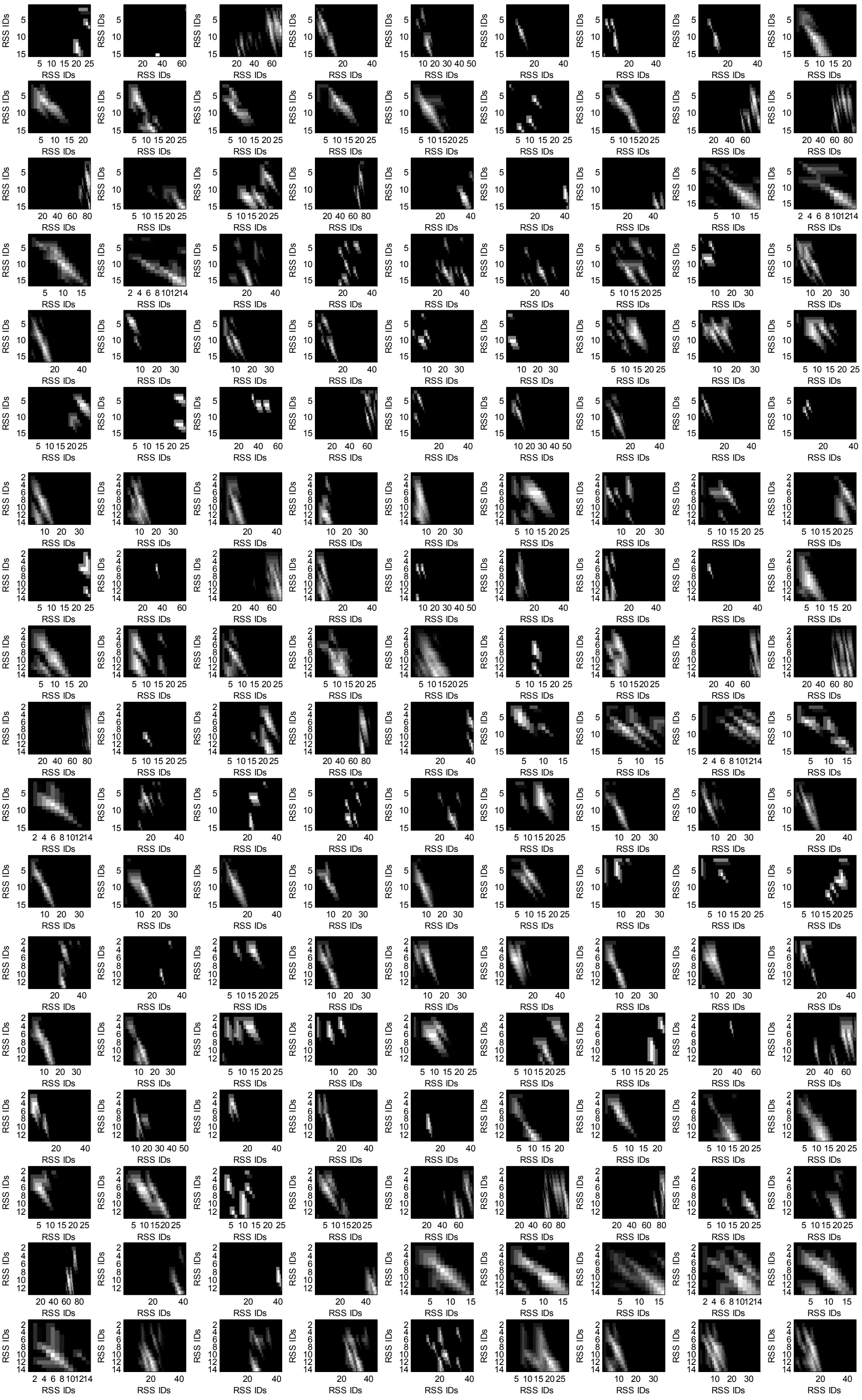
RSS IDs

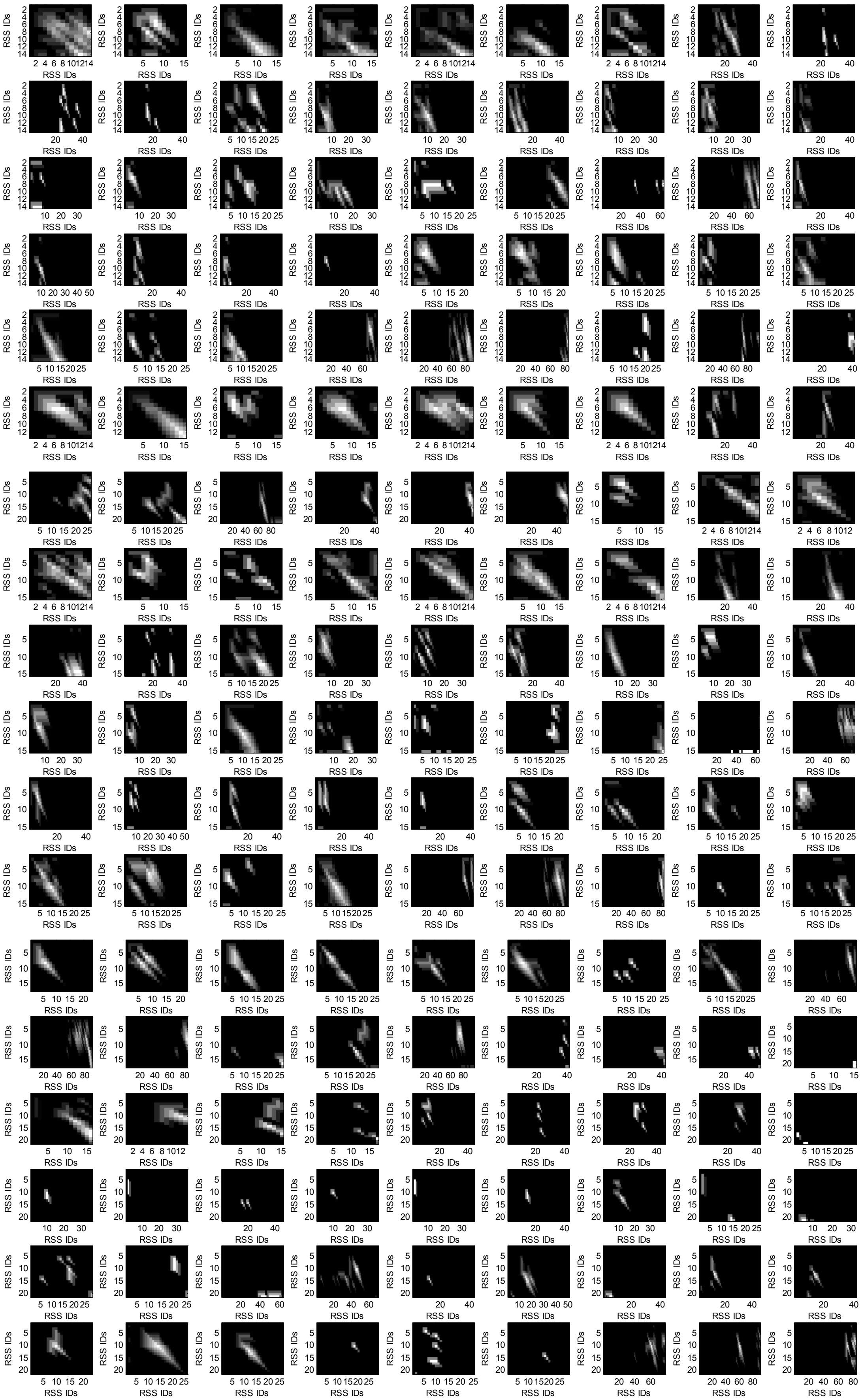
RSS IDs

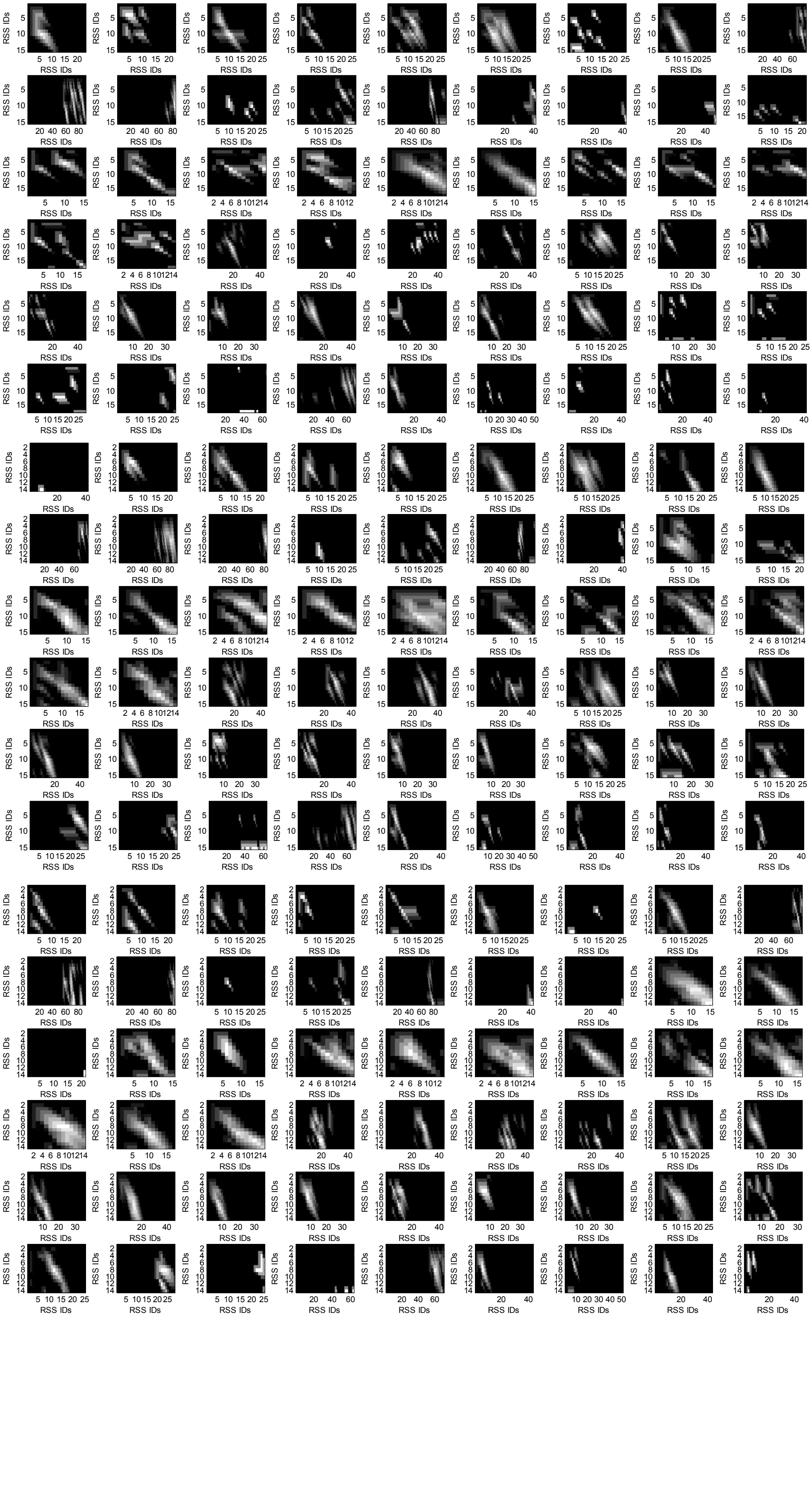
RSS IDs

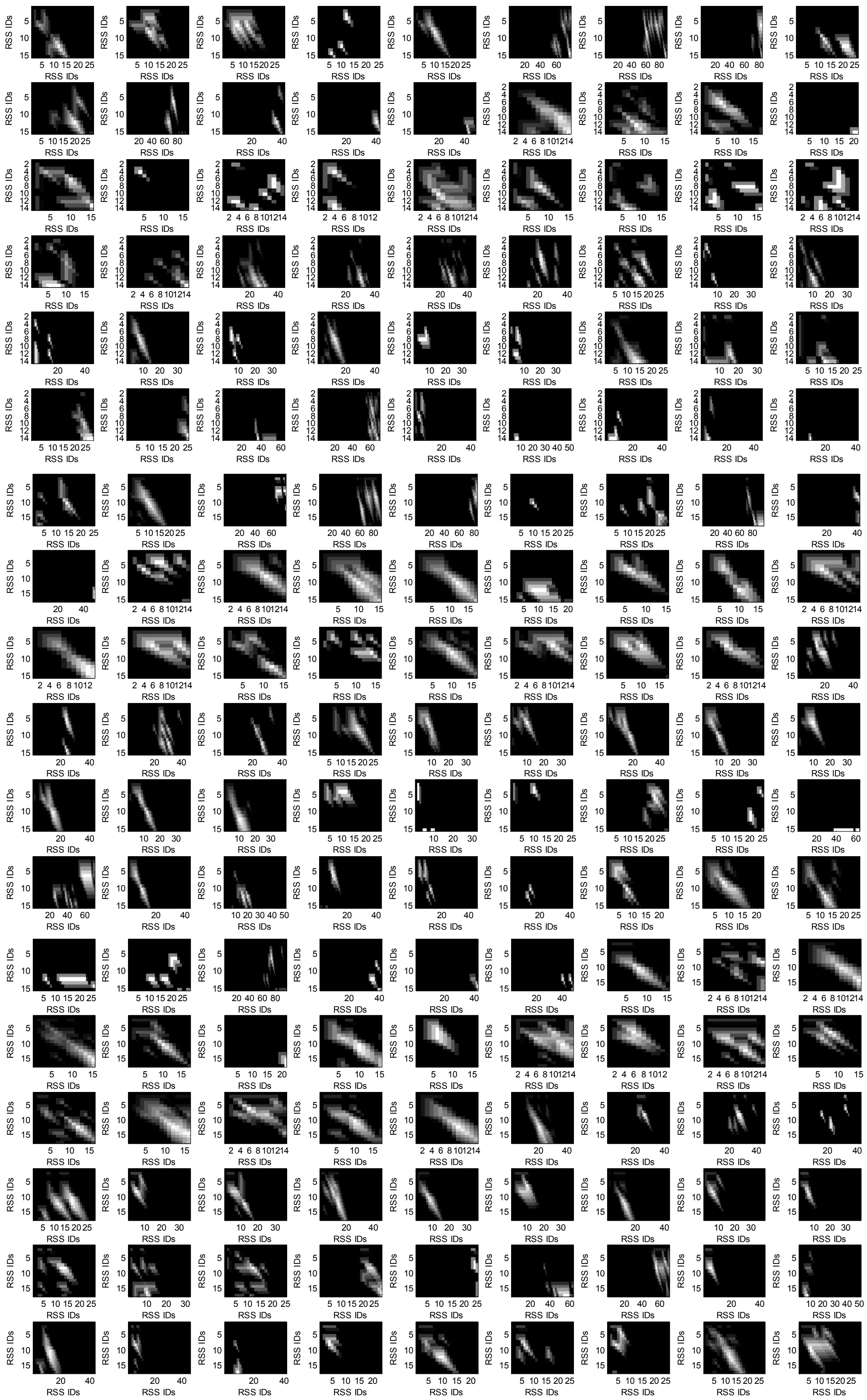
RSS IDs

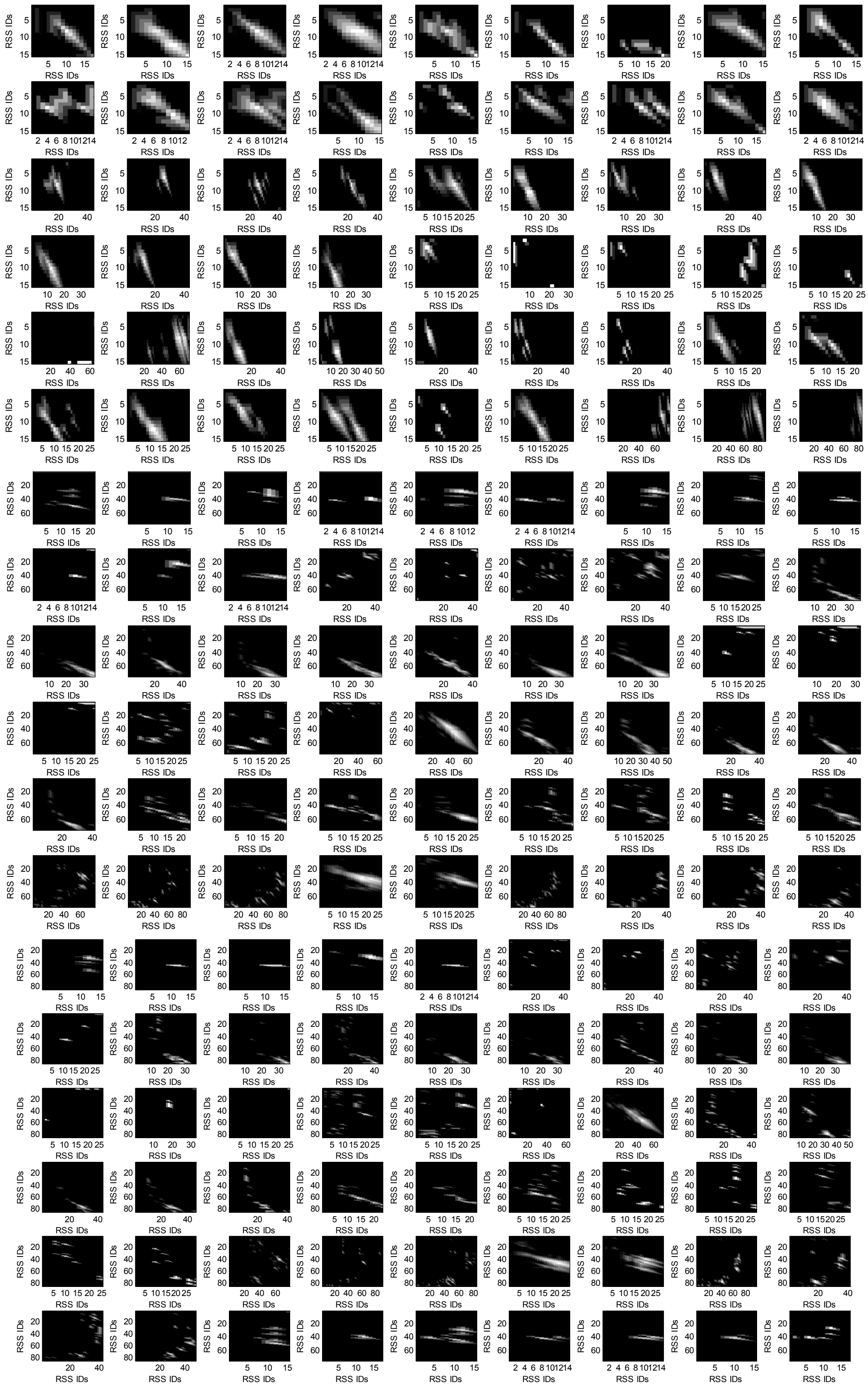
RSS IDs

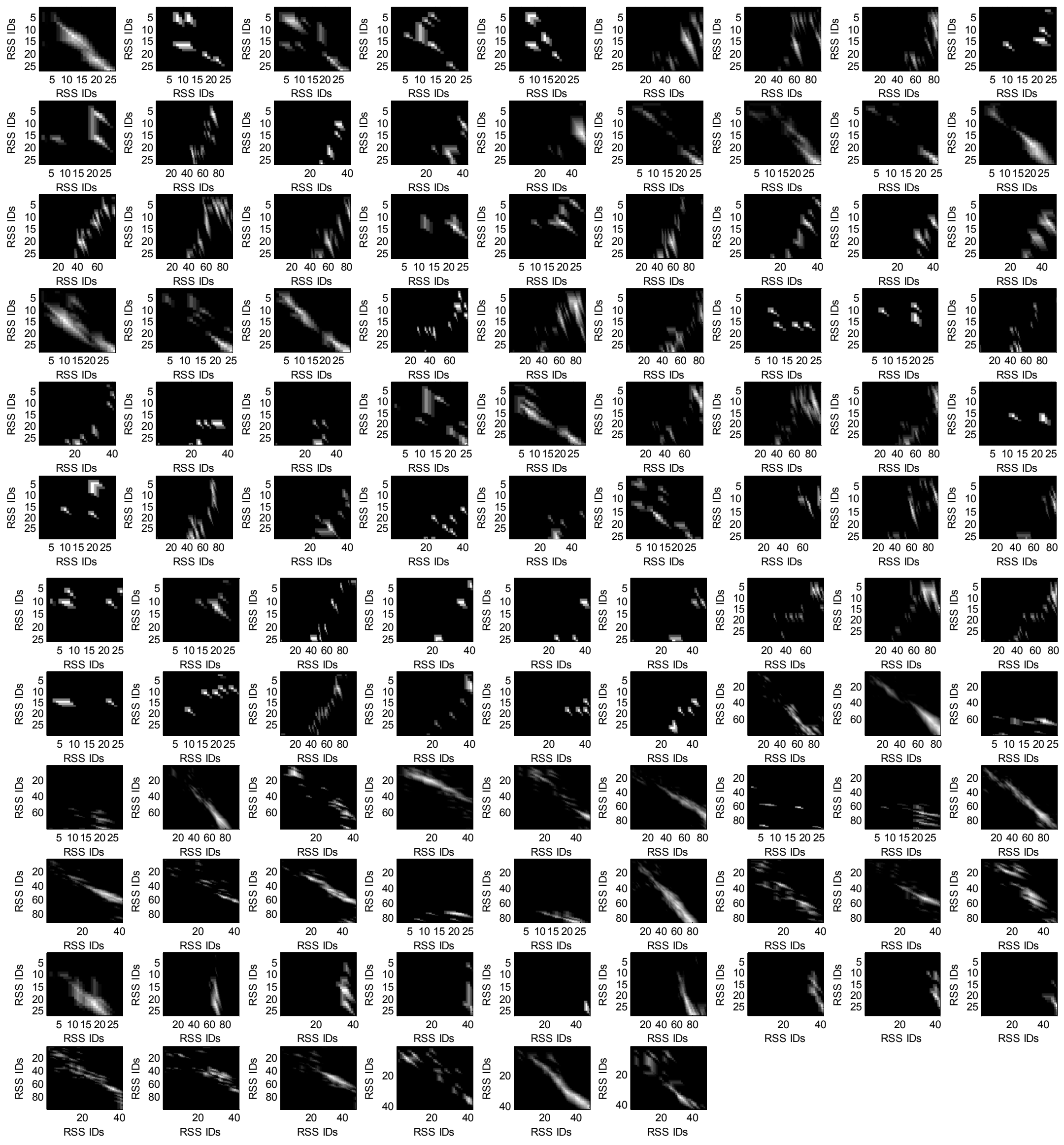

© 2015 by the authors; licensee MDPI, Basel, Switzerland. This article is an open access article distributed under the terms and conditions of the Creative Commons Attribution license (http://creativecommons.org/licenses/by/4.0/).
